# Sub-annual resolution evidence for limited impact of the 74-ka Toba eruption on eastern African climate

**DOI:** 10.1126/sciadv.adv6851

**Published:** 2026-07-31

**Authors:** Jinheum Park, Christine S. Lane, Philip A. Barker, Christine Cocquyt, Maarten van Daele, Maxim Deprez, Yvonne Hamann, Jack H. Lacey, Melanie J. Leng, Catherine Martin-Jones, Clive Oppenheimer, Céline-Marie Vidal, Christian Wolff, Dirk Verschuren

**Affiliations:** ^1^ Department of Geography, University of Cambridge; Cambridge, CB2 3EN, United Kingdom.; ^2^ Lancaster Environment Centre, Lancaster University; Lancaster, LA1 4YQ, United Kingdom.; ^3^ Research Department, Meise Botanic Garden; Meise, B-1860, Belgium.; ^4^ Limnology Unit, Department of Biology, Ghent University; Gent, B-9000, Belgium.; ^5^ Renard Centre of Marine Geology, Department of Geology, Ghent University; Gent, B-9000, Belgium.; ^6^ Centre of X-ray Tomography-PProGRess, Department of Geology, Ghent University; Gent, B-9000, Belgium.; ^7^ Max Planck Institute for Chemistry, Climate Geochemistry; Mainz, 55128, Germany.; ^8^ National Environmental Isotope Facility, British Geological Survey; Nottingham, NG12 5GG, United Kingdom.; ^9^ Centre for Environmental Geochemistry, School of Biosciences, University of Nottingham; Nottingham, LE12 5RD, United Kingdom.; ^10^ Istituto Nazionale di Geofisica e Vulcanologia, Osservatorio Etneo; Catania, 95125, Italy.; ^11^ Fitzwilliam College; Cambridge, CB3 0DG, United Kingdom.

## Abstract

The “super-eruption” of Mt. Toba in Indonesia ∼74,000 years ago, probably the largest volcanic event witnessed by humanity, has stimulated decades of research into the eruption’s impacts on global climate and ancient human ecology. Progress has been hampered by lack of geological proxy records covering the critical time window with sufficient resolution to discern the environmental impacts directly due to the eruption. Here, we present sub-annually resolved proxy data from eastern tropical Africa which constrain the timing of the Toba eruption to Northern Hemisphere winter and suggest that it triggered modest cooling and acute drought lasting less than two years. This short-lived climate perturbation was superimposed on a multi-decadal cooling/drying trend coeval with a phase of northern high-latitude cooling characterizing the world’s transition into the last glacial period. Our findings indicate that climatic impacts of the Toba eruption in eastern Africa were modest compared with other natural climate dynamics, henceforth allowing improved parameterization of earth-system models assessing its environmental impact.

## Teaser

Sub-annually resolved lake sediments suggest the Toba eruption caused modest cooling and drought in eastern Africa.

## INTRODUCTION

The Youngest Toba Tuff (YTT) “super-eruption” of Mt. Toba in Sumatra, Indonesia ∼74,000 years ago (73.7 ± 0.3 kyr ago) (*1*) is widely regarded as the largest volcanic event of the Quaternary period (the last 2.6 million years). Estimates of the volume of ash it erupted over a 9–14 day period (*2*) range between 2800 and 5,300 km^3^ dense-rock equivalent (*3*, *4*), three orders of magnitude larger than the 1991 eruption of Mt. Pinatubo in the Philippines (*5*), the largest eruption in modern times. Capturing attention from paleoclimatologists and paleoanthropologists for decades, some studies postulated that the stratospheric veil of sulfate aerosol produced by the Toba eruption accelerated the world’s transition from the last interglacial period (Marine Isotope Stage 5; MIS5) to the full-glacial conditions of MIS4 (*6*), or of a millennial-scale phase of northern-hemisphere cooling preceding this transition (*7*, *8*). The implied idea that this eruption triggered a global “volcanic winter” in turn fueled arguments that it may have resulted in the near-extinction of (*9*, *10*) or behavioral adaptations among (*11*) contemporary populations of *Homo sapiens* in eastern Africa.

Contrasting with this paradigm of major environmental impact, model simulations of the Toba eruption suggest a relatively modest and ephemeral climatic perturbation (*12*, *13*). However, these model outcomes are strongly predicated on the amount of sulfur (S) thought to have been injected into the stratosphere, estimates of which range widely between 100 and 9000 Tg (*12*, *14*, *15*); as well as on their consideration of aerosol microphysics for very large S releases, which lead to self-limiting effects on radiative forcing and hence the magnitude of post-eruption temperature and hydroclimate effects (*12*, *13*). Other uncertainties in model output reflect weak constraints on the altitude and dispersal of the aerosol veil, the time of the year of the eruption, and on the prevailing climate sensitivity (*12*, *16*, *17*).

**Fig. 1.**
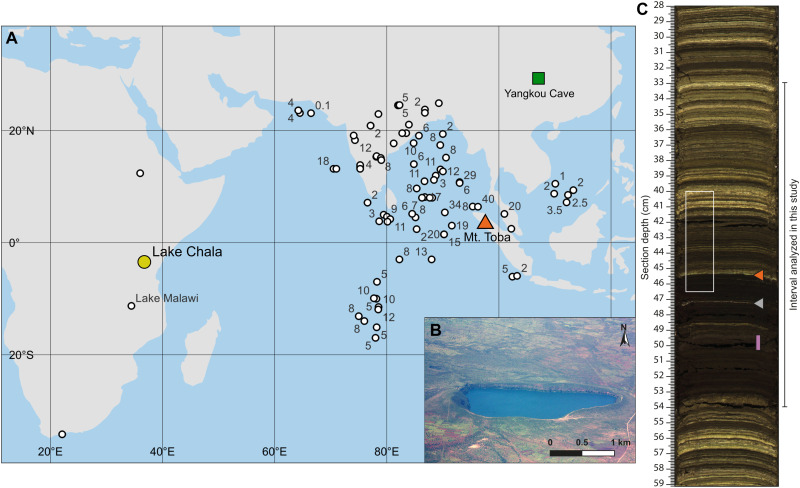
Geographical and stratigraphical setting of the ∼73.7-ka Toba eruption. (**A**) Locations of Lake Chala (yellow circle), Yangkou Cave (green square), and the Mt. Toba volcanic caldera (orange triangle) in relation to other documented occurrences of Youngest Toba Tuff (YTT) tephra in marine and continental settings (white circles) with local thickness of the ashfall deposit in cm. Map generated using Natural Earth, open-source vector and raster map data in QGIS ver. 3.40.12. (**B**) Oblique aerial view of Lake Chala within its steep-sided crater basin, viewed from the south (Photo Credit: Hansbaer, Wikimedia Commons; CC BY-SA 3.0; https://creativecommons.org/licenses/by-sa/3.0). Due to this highly restricted catchment, the sedimentary distribution of YTT glass shards (Fig. 2A) mostly represents direct ashfall onto the lake surface rather than delayed input from crater runoff. (**C**) Line-scan image of core section DCH-CHL16-1B-21H-2 with indication of the ∼450-year time window analyzed in this study. The white box encloses the 6.5-cm interval displayed in Fig. 2A. The orange and gray triangles show the stratigraphic positions of the YTT cryptotephra (DCH-67.55) (*28*, *29*) and a 1-mm-thick visible tephra (DCH-67.56) originating from a volcano in the East African Rift System (*29*), respectively. The purple bar indicates the position of a layer of predominantly clastic material flushed into the lake from catchment soils (see text and Fig. 3, D to H).

Whereas models provide valuable explanations of process, the actual climatic and environmental impacts of the Toba eruption can only be assessed through investigation of geological archives containing YTT ashfall (tephra). YTT ashfall has been identified in marine sediment cores recovered from offshore Sumatra (*18*), the South China Sea (*19*), the Bay of Bengal (*20*) and the Arabian Sea (*21*), as well as in terrestrial sedimentary sections in India, Malaysia and South Africa (*20*, *22*, *23*) and (ancient) lake deposits in eastern Africa (*11*, *24*–*26*) (Fig. 1A). However, most paleoenvironmental reconstructions reported hitherto have attained at best multidecadal temporal resolution (*20*–*22*), and even the sub-decadal resolution provided by sediment cores from Lake Malawi (*24*–*26*) and the northern Arabian Sea (*27*) are insufficient to resolve the climate impacts at interannual scale typically associated with volcanic forcing (*12*, *13*). Another issue with paleoenvironmental proxies is that post-Toba vegetation changes documented at some sites in India (*20*, *22*) may not reflect eruption-forced climate change but rather the direct impacts of ash deposition. Further, the timing of the Toba eruption during or near the MIS5–MIS4 transition adds to the challenge of detecting the signature of its climatic impact against a background of progressive global cooling.

**Fig. 2.**
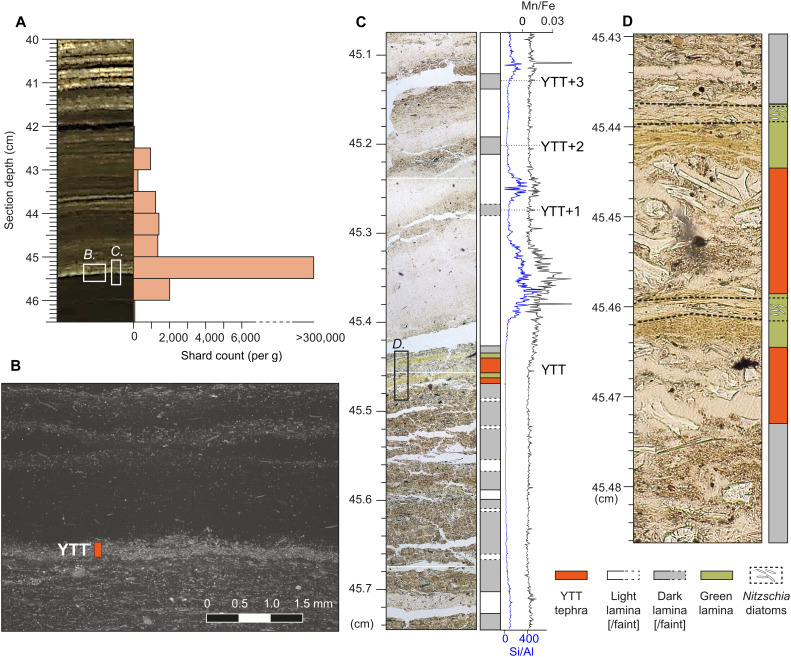
Detailed view of Lake Chala sediments containing the YTT ashfall deposit. (**A**) Partial line-scan image of DCH-CHL16-1B-21H-2 with bar graph showing the abundance, in contiguous 0.5-cm increments, of volcanic glass shards geochemically characterized as YTT ashfall (*28*). (**B**) Micro-computed tomography (μCT) image of the 4-mm interval at 45.55–45.15 cm depth centered on the ∼0.3-mm thick YTT glass-shard deposit, distinct from organic mud below (light hue in the image) and diatoms above (dark hue). (**C**) Microscopic thin-section image and schematic microstratigraphy of Lake Chala sediments bridging the YTT ashfall deposit, and corresponding high-resolution Si/Al and Mn/Fe profiles measured along a parallel line by inductively coupled plasma mass spectrometry (ICP-MS; see also fig. S2, B and C). The labels YTT + 1, YTT + 2 and YTT + 3 besides dark varve laminae refer to the number of years after Toba ashfall. Varve boundaries in the time interval before YTT ashfall are approximate due to the faint character of light laminae. The thin-section image is assembled from four separate photographs, their boundaries indicated by thin horizontal white lines. Despite resin-filled spaces formed during thin-section preparation, the original sediment structure is preserved. (**D**) Close-up of the YTT ashfall deposit showing the two featureless light-green layers within and above the glass-shard layer, and associated clusters of *Nitzschia* diatoms. Rectangles in (A) and (C) are indicative of the stratigraphic depth ranges shown in (B) to (D), but not of their exact planar positions due to sampling or imaging constraints.

Here, we present the first sub-annually resolved reconstruction of climate conditions and environmental variability in the immediate aftermath of the Toba eruption, based on multi-proxy analysis of the annually-laminated (varved) sedimentary record of Lake Chala in eastern equatorial Africa (Fig. 1, A to C), which contains an undisturbed deposit of primary YTT ashfall (Figs. 1C and 2) (*28*, *29*). Focusing on the ∼450-year sedimentary sequence bridging this YTT deposit (Supplementary Materials), our reconstruction draws on the microstratigraphy of successive varve laminae, micro x-ray fluorescence (μXRF) scanning and inductively-coupled plasma mass spectrometry (ICP-MS) of mineral-element composition at sub-annual resolution, supplemented by stable carbon and nitrogen isotopic measurements on bulk organic matter, and analysis of fossil diatom assemblages at 2–3 year resolution (Materials and Methods). Combined, these data reflect multiple aspects of Lake Chala’s system-level response to the climatic disturbance caused by the Toba eruption in the Lake Chala area, and by extension in the greater Horn of Africa region where monsoonal rainfall is sourced exclusively from the Indian Ocean (*28*).

The Lake Chala sediment sequence was drilled in 2016 by the DeepCHALLA project (*30*) (Materials and Methods) and spans the last ∼250 kyr (*29*, *31*). In this continuous sequence of deepwater lacustrine deposits, alternating dark and light sediment laminae form annual couplets (i.e., varves) (Materials and Methods) (*28*). Dark laminae contain abundant organic matter derived from phytoplankton (mostly green algae and cyanobacteria) (*32*) mixed with terrestrial components eroded from the crater (Fig. 1B). They form under conditions of generally strong water-column stratification prevailing from October to May, spanning the “short rains” (October–December) and “long rains” (March–May) as well as the intervening warm dry season (January–February). The light laminae consist mostly of fossil diatoms, and represent the annual diatom bloom that develops during the main dry season (June–September) (*33*, *34*). In this time of year coincident with Southern Hemisphere (austral) winter, lake-surface cooling and monsoonal winds generate deeper mixing of the lake (*35*), bringing nutrients to the surface (*32*, *36*) and enabling diatoms to remain afloat within the photic upper water column (*33*). At the interannual time scale over the past century, thick light laminae generally formed when eastern equatorial Africa experienced a prolonged dry season due to slightly cooler conditions characterizing the La Niña phase of the El Niño-Southern Oscillation (ENSO), whereas thin light laminae (weaker diatom blooms) formed during slightly warmer El Niño years when eastern equatorial Africa experienced wetter conditions (*33*) (Materials and Methods). Demonstration of this connection (*33*) highlights the ecological sensitivity of Lake Chala to high-frequency/low-amplitude climate variability, as well as the capacity of its sediment record to register such short-lived, modest climate anomalies through the set of lake-status proxies analyzed in this study.

## RESULTS

### Primary ashfall of the Toba eruption in eastern equatorial Africa

The four confirmed YTT deposits in Africa (*11*, *23*, *24*, *28*) (Fig. 1A) represent the most distal occurrences of ash from the Toba eruption found to date. In the Lake Chala record, peak concentrations of YTT glass shards (∼300,000 g^−1^ dry sediment) occur between 45.5 and 45.0 cm depth in core section DCH-CHL16-1B-21H-2 (67.55–67.54 meter composite depth, mcd), with a tail of strongly reduced shard concentrations extending upward to 42.5 cm depth (Fig. 2A) (*28*). Very few glass shards occur in the mud immediately below this peak (Fig. 2A), indicating minimal vertical displacement of the shards following their settling from the water column. A micro computed-tomography (μCT) image of the 4-mm core interval centered on the ∼0.3-mm-thick YTT deposit emphasizes its distinct lower and upper boundaries (Fig. 2B), indicative of primary ashfall onto the lake surface rather than delivery via runoff from the crater catchment. In an epoxy-hardened microscopic section of the bracketing interval (Materials and Methods), the YTT deposit appears as a distinct ∼0.3-mm-thick layer of glass shards, located within but close to the upper boundary of a ∼1.2 mm-thick layer of dark organic mud (Fig. 2, C and D).

### Sedimentary signatures of Toba-induced climate perturbation

Our combined sedimentary proxies indicate that during the ∼260-year period prior to the Toba eruption (assuming a mean sedimentation rate of 0.33 mm year^−1^ between 53.0 cm and 45.4 cm section depth; Supplementary Materials), the Lake Chala region experienced relatively wet climate conditions. Infrequent and poorly-developed light laminae (Figs. 1C and 2) reflect limited diatom production, implying a water column that remained strongly stratified year-round (*34*), and thus a generally positive lake water balance and limited surface cooling or wind stress (*33*).

**Fig. 3.**
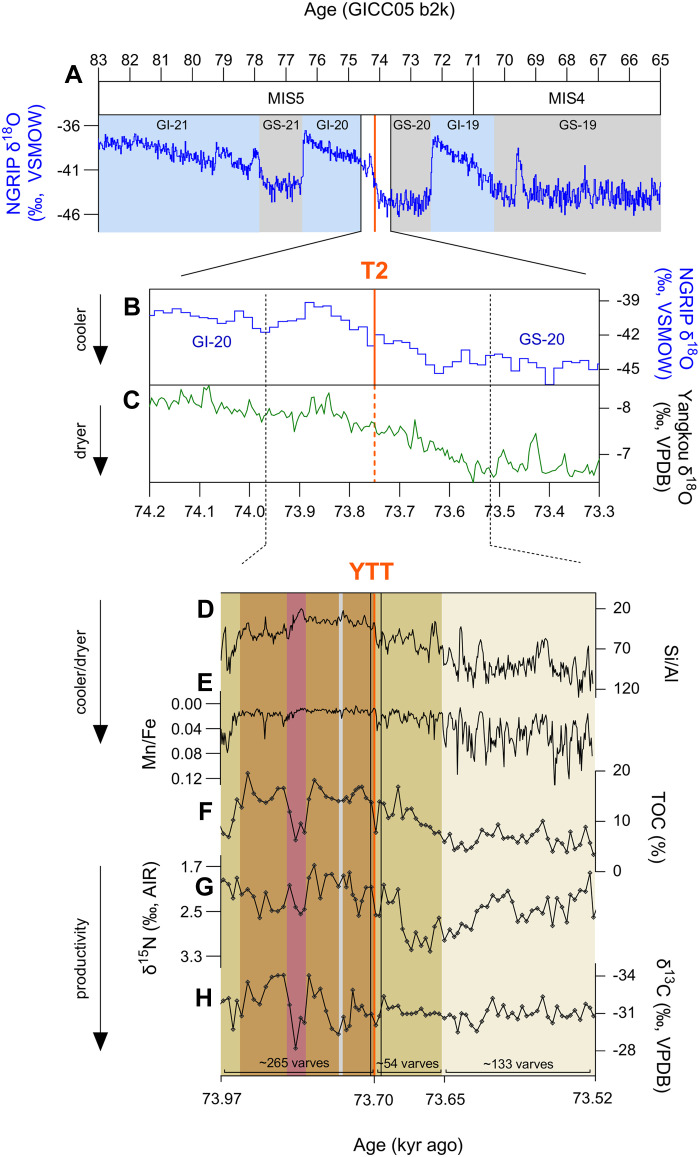
Global context of climate change in eastern Africa before and after the Toba eruption. (**A**) Oxygen isotope (δ^18^O) variation in Greenland ice (*50*, *75*, *76*) between 83 and 65 kyr ago highlighting the position of sulfate peak T2 attributed to the Toba eruption (*8*) (bold orange line) in relation to the succession of warm (Greenland Interstadial, GI) and cool (Greenland Stadial, GS) phases around the start of the last glacial period ∼71 kyr ago (Marine Isotope Stage 5 (MIS5) to MIS4 transition; (*77*). (**B**) Close-up of the 800-year central section of (A) on the AICC2012 time scale (*78*) where T2 is placed at 73.75 kyr ago (*47*). (**C**) Speleothem δ^18^O variation at Yangkou Cave in southern China (*51*), reflecting relative strength of the East Asian Summer Monsoon (EASM) during the equivalent 800-year time window (*47*). (**D** to **H**) Climate-driven environmental change in Lake Chala during the ∼450-year period bridging the local YTT ashfall deposit (bold orange line), with absolute ages based on varve counting relative to the YTT deposit which is nominally fixed at 73.70 kyr ago. The background shading reflects general sediment color (Fig. 1C), with purple and gray vertical bars indicating the stratigraphic positions of the flushed soil deposit and tephra DCH-67.56, respectively (Fig. 1C). The thin black vertical lines on both sides of YTT bracket the 45.7–45.1 cm interval shown in Fig. 2C. [(D) and (E)] Elemental ratios Si/Al and Mn/Fe (measured by micro-x-ray fluorescence; μXRF) reflecting, respectively, variations in fossil diatom abundance and the depth of water-column mixing. Note the reversed scale, with higher values indicative of cooler/drier climatic conditions. (F) Total organic carbon (TOC) content. [(G) and (H)] Nitrogen (δ^15^N) and carbon (δ^13^C) isotopic composition of the organic component.

The Si/Al and Mn/Fe ratios of these sediments are strongly correlated (*r* = 0.777, *P* < 0.0001, *n* = 429; fig. S1). Si/Al is a proxy for the abundance of fossil diatoms versus clastic mineral matter, which here reflects the relative prominence of dry-season diatom blooms versus wet-season soil run-off. Mn/Fe represents variability in average water-column redox conditions, which are linked closely to the depth of mixing because mixing injects oxygen into the water column which in turn oxidizes (and precipitates) dissolved Mn^2+^ more readily than Fe^2+^ (*34*). Strong correlation between Si/Al and Mn/Fe in the analyzed section of the Lake Chala record implies broad proportionality between the magnitudes of climatic cooling (causing deeper-than-average mixing and nutrient recycling) and drying (limiting wet-season soil run-off) controlling these two lake-system proxies. Sustained low values of Si/Al and Mn/Fe in the period before the Toba eruption (Figs. 2C and 3, D and E) point to limited mixing of the water column creating unfavorable conditions for diatoms to compete with other algae, combined with greater soil run-off and non-diatom phytoplankton production; and thus, a relatively warm and wet climate regime.

A ∼1-cm-thick layer of mineral-rich sediment ∼5 cm below the YTT horizon (50.5–49.5 cm; purple bar in Fig. 1C), characterized by low organic content and elevated carbon isotopic ratio (Fig. 3, F and H) represents a pulse of eroded crater slope material, most likely flushed into the lake by an exceptionally heavy rainfall event. The 1-mm-thick visible tephra located at ∼47.2 cm (DCH-67.56; gray triangle in Fig. 1C) can be sourced to a volcanic eruption within the East African Rift System (*29*) (Supplementary Materials). As detailed below, proxy evidence of the ecological perturbation of Lake Chala associated with these two events helps constrain the regional climatic impact of the Toba eruption.

Under high-magnification light microscopy, the YTT glass-shard horizon in Lake Chala lies immediately above dark mud interspersed by thin diatom layers which are often poorly defined (Fig. 2C) and equivalent to the faint white laminae that punctuate the section of dark mud below YTT in visible stratigraphy (Fig. 1D). Notably, the YTT ashfall deposit is interrupted by a thin (∼0.04 mm) layer of featureless light-green organic material and capped by a similar light-green layer, before being overlain by a thin (∼0.07 mm) lamina of dark mud (Fig. 2D). We interpret these amorphous light-green layers, containing no visible algal or microbial remains, as deposits of extracellular polymeric substances (EPS), organic compounds which diatoms produce under acute stress (*37*). Here, EPS production might point to reduced light conditions caused by the YTT ash and aerosol veil (*13*), which may have been most effective for 1–2 months until poleward transport of the aerosol veil (*38*–*40*). This would have compounded the already difficult conditions for diatom growth in Lake Chala due to persistently poor nutrient conditions associated with strong lake-column stratification. Notably, no other such clear light-green layers occur in the entire section of dark mud below the YTT deposit. Within and above each of the two EPS layers directly associated with YTT, occasional *Nitzschia* occur (Fig. 2D), evidence of modest diatom production and hence indicative of at least intermittent water-column mixing (*32*), possibly triggered by the immediate surface cooling effect of the Toba aerosol veil.

The thin dark lamina overlying the upper EPS layer is followed by a ∼1.2-mm-thick light-colored lamina representing the first post-Toba main dry season (Fig. 2C). Markedly elevated Si/Al and Mn/Fe values (Fig. 2C; fig. S2, B and C) and abundant *Nitzschia* diatoms (fig. S2D) indicate a dry season characterized by exceptionally deep and/or prolonged water-column mixing, as would occur when aerosol-induced radiative cooling of the lake surface further reduced the already low thermal gradient through the water column typical of cool-season limnological conditions (*32*, *36*) (Materials and Methods). Importantly, the weakly pronounced dark varve lamina overlying this thick diatom layer (Fig. 2C) indicates that deep mixing persisted well into the following “short rains” season. Representing around eight months of mostly wet weather conditions and strong water-column stratification coincident with austral summer (*32*–*34*, *36*), the modest thickness (∼0.1 mm) and faint appearance of this dark lamina indicates limited surface catchment run-off (hence, below-average rainfall) coupled with relatively low non-diatom phytoplankton production.

### Duration of Toba-induced climate and ecosystem impacts in eastern Africa

Deposition of a ∼1.2-mm-thick light lamina shortly above the main YTT glass-shard horizon, following a ∼260-year interval with near-absence of light laminae testifying to limited diatom production only, represents an abrupt shift in the ecological functioning of Lake Chala synchronous with the atmospheric dispersal of YTT aerosol. Reflecting a period of prolonged and/or exceptionally deep water-column mixing, this prominent dry-season signature in the year after the eruption (i.e., during YTT + 1; Fig. 2C) was most likely caused by eruption-induced additional cooling of the region’s continental atmosphere during austral winter. Also, the poorly expressed YTT + 1 dark lamina, reflecting comparatively weak post-Toba rain seasons, can be explained by monsoonal moisture advection to Lake Chala being compromised by eruption-induced atmospheric cooling over western Indian Ocean source regions (*41*). Notably, light-colored varve laminae thin again after the first post-Toba year (i.e., during YTT + 2 and YTT + 3), while dark laminae become better expressed resulting in overall more distinct and regular alternation of light and dark laminae (Figs. 1C and 2C). Together, this succession of post-YTT light and dark laminae indicates that the acute regional climate response to the Toba eruption lasted at most until the “long rains” wet season in YTT + 2. This is supported by the abrupt switch to lower Mn/Fe values above the robust YTT + 2 dark lamina (Figs. 2C and 3E), indicating a return to more sustained water-column stratification during the third post-Toba year (leading up to deposition of the YTT + 3 dark lamina).

Bulk organic matter deposited in the first two varve couplets above the YTT horizon (i.e., during YTT + 1 and YTT + 2) features elevated carbon (δ^13^C) and nitrogen isotope (δ^15^N) values (Fig. 3, G and H, and fig. S2, F and G). Most likely these reflect the enhanced photosynthetic fractionation which must have occurred during the massive and prolonged blooming of diatom algae in the first post-Toba dry season (*42*). We exclude the alternative explanation that they represent a terrestrial vegetation response to Toba-induced drying (i.e., a shift to more C4 plants such as grasses), considering the short time interval covered by these samples (2–3 years; fig. S2) and the predominantly aquatic origin of organic matter in Lake Chala sediments (*32*). We also rule out the possibility that the elevated δ^13^C and δ^15^N values reflect Toba-induced fertilization, given the low quantity of Toba ashfall onto Lake Chala (only 0.3 mm thick, the YTT deposit is not macroscopically visible; Fig. 1C) and the lake’s naturally high dissolved silica content (*42*). Moreover, no strong diatom bloom (Si/Al; Fig. 3D) nor a change in water-column mixing (Mn/Fe; Fig. 3E) occurred after fallout of the 1-mm thick tephra DCH-67.56 (gray bar in Fig. 3, D to H) a few decades prior to the Toba eruption, even though this local eruption (*29*) delivered several orders of magnitude more glass shards than Toba. The strongly reduced organic carbon content (TOC) immediately above the YTT horizon (Fig. 3F and fig. S2E) can be attributed to high diatom abundance (peak Si/Al values; Fig. 2C and fig. S2A), given that the organic component of diatom cells is readily oxidized during postmortem descent through the water column, so that fossil diatom cells typically contain <1% TOC (*43*).

## DISCUSSION

### The Toba eruption occurred during Northern Hemisphere winter

The time of year (or local season) when a large low-latitude volcanic eruption such as Toba occurred has implications for its global climatic impact (*12*, *17*, *44*). Based on the distribution of ash fallout, it has been suggested that the Toba eruption occurred during Northern Hemisphere summer (*45*). However, sedimentary sequences which span the eruption’s timeframe and have captured its ash in locations distant from Indonesia are scarce (Fig. 1A), hampering the full mapping of ash dispersal required to verify ash distribution models (*4*). The sub-annually resolved Lake Chala record uniquely allows us to establish the time of year of the Toba eruption.

The distinct YTT horizon occurs close to the upper boundary of a dark varve lamina (Fig. 2, C and D), implying that it was deposited in the latter half of the period between October and May. In addition, the few *Nitzschia* diatoms enclosed within the EPS layers directly associated with YTT (Fig. 2D) are indicative of the modest nutrient upcycling which occurs during the short dry season in austral summer (January–February) when warmed-up surface water limits deep mixing (*36*) (Materials and Methods). Our data therefore indicate that the Toba eruption occurred during Northern Hemisphere (boreal) winter (Supplementary Materials). By favoring dispersal of aerosol into the Northern Hemisphere (*38*), this timing would have resulted in greater globally-averaged cooling by radiative forcing (*38*), and particularly so over the large Northern Hemisphere landmasses (*12*, *44*). Equatorial Africa is less sensitive to this seasonal influence (*38*) but may have experienced cooling via atmospheric teleconnection to northern high-latitude climate dynamics.

### Embedding Toba’s climatic impact in global climate variability ∼74 kyr ago

Our combined proxy data from immediately above the YTT horizon, indicating that the direct climatic impact of the Toba eruption in eastern Africa did not likely exceed ∼18 months (until austral winter halfway through YTT + 2) are compatible with climate model simulations in which precipitation over this region recovers (even slightly increases) within two years after a volcanic super-eruption (*12*, *46*), notwithstanding that these models use prescribed sulfur ejections of 850–1000 Tg S, i.e. much larger than current ice-core based estimates for the amount of sulfur ejected by Toba (∼200 Tg S) (*8*, *47*). Zooming out to longer-term climate variability in eastern Africa ∼74 kyr ago as registered in the full ∼450-year time window covered by our analysis (Fig. 3, D to H) provides a means to also constrain the magnitude of eastern African cooling caused by the Toba eruption.

Marked alternation of dark-lamina-dominated and light-lamina-dominated mud in the studied section of the DeepCHALLA climate archive (Fig. 1C) reflects a succession of relatively wet and relatively dry regional climate conditions at decadal-to-centennial time scales (*33*), as also at present typifies dry (arid to sub-humid) regions of eastern Africa (*48*); and onto which the short-lived and comparatively modest local climatic imprint of the Toba eruption was superimposed. Specifically, the ∼1.2-mm thickness of the first post-Toba light lamina is not unusual compared to other light laminae deposited in the decades after the eruption (Fig. 1C). The common prevalence of thick light laminae upwards of 41.6 cm depth in DCH-CHL16-1B-21H-2 (Fig. 1C), associated with high Si/Al, high Mn/Fe and low TOC values (Fig. 3, D to F), indicates that the Toba eruption occurred as eastern equatorial Africa transitioned from a relatively wet (and slightly warmer) to a drier (and slightly cooler) climate regime, or at least a climate regime with more pronounced dry season. The exact timing of this climatic transition relative to the Toba eruption is not readily ascertained from our lake-system data, as it is proxy-dependent (Fig. 3: compare panels D to F). However, the magnitude of regional climate perturbation caused by the Toba eruption clearly did not exceed natural climate variability in the ∼450-year period covered by our high-resolution analyses, and is certainly unexceptional compared with climate variability in eastern Africa across glacial-interglacial timescales (*28*).

In polar ice cores from both Greenland and Antarctica, a peak in sulfate deposition identified as T2 and dated to 74.161–74.154 kyr b2k (before 2000 CE; Fig. 3, A and B) (*8*, *49*) has been tentatively attributed to the Toba eruption on the basis of its amplitude, timing and bipolar distribution, but lacks a confirmatory deposit of YTT glass shards (*8*, *47*). Synchronizing T2 with the YTT deposit in Lake Chala places our evidence for Toba-triggered climate and environmental change over eastern Africa in a global context. The Toba eruption occurred ∼4000 years before the end of the last interglacial period (as defined by the MIS5–MIS4 boundary ∼71 kyr ago; *50*) (Fig. 3A) at a time when northern high-latitude (or more broadly, northern extratropical) regions were transitioning from relatively warm climate conditions during Greenland Interstadial 20 (GI-20) to cooler Greenland Stadial 20 (GS-20) (Fig. 3, A and B) (*50*). Speleothem oxygen-isotope (δ^18^O) records from Yangkou Cave in southern China (Fig. 1A) covering the equivalent time window (*51*) show a more gradual but broadly coeval trend towards drier climate conditions (Fig. 3C), attributed to weakening of the East Asian Summer Monsoon as manifested by reduced moisture advection onto a colder Eurasian continent (*52*, *53*). Assessing the exact distribution of climate anomalies in high- and low-latitude regions at this time scale is beyond the scope of this study. However, given robust evidence for northern high-latitude forcing of millennial-scale hydroclimate variation in (sub-) tropical regions worldwide (*54*, *55*), we highlight the temporal match between the progressive cooling/drying trend in eastern equatorial Africa spanning the YTT ashfall deposit in Lake Chala (Fig. 3, D to F) and the progressive Northern Hemisphere cooling (Fig. 3, A and B) and drying (Fig. 3C) registered before and after the T2 sulfate peak in Greenland ice. Taking into account the overall positive relationship between moisture balance and temperature in eastern equatorial Africa during the last glacial period (*28*), our data from the GI-20/GS-20 transition period suggest that, with regard to millennial-scale climate events punctuating the incipient phase of the last glaciation, Northern Hemisphere cooling extended to the equator already before the end of MIS5.

### Estimating the magnitude of Toba radiative cooling

The chronologically tight link between the Chala and Greenland climate archives, enabled by the shared YTT-T2 time marker (Fig. 3), provides an important constraint on the absolute magnitude of cooling in equatorial Africa potentially caused by the Toba eruption. Importantly, the magnitudes of regional cooling and drying caused by the Toba eruption are intrinsically linked, by the fact that the direct aerosol-induced radiative cooling which caused deeper-than-usual dry-season mixing in Lake Chala must also have cooled surface-ocean waters in the western Indian Ocean which are the moisture source of eastern African rainfall. Reduced surface-ocean evaporation and weakened monsoonal moisture advection onto land together thwarted the post-Toba rain seasons as evidenced by our data. If the cooling of eastern Africa registered in the latter half of our proxy time series (i.e., unrelated to the Toba eruption but contemporaneous with the GI-20/GS-20 transition in northern extratropical regions; Fig. 3) had exceeded ∼2°C, as occurred during the Last Glacial Maximum and early Lateglacial period (MIS2) (*56*), it would have forced complete mixing of Lake Chala and have generated bottom turbulence creating cm-scale banded sediments (*28*) unlike the varved sediments deposited in the latter part of the studied time window (Fig. 1C). Assuming broad proportionality between the magnitudes of Si/Al and Mn/Fe excursions in our proxy time series and the magnitude of cooling causing deep mixing in Lake Chala, it follows that the Toba-induced radiative cooling cannot have exceeded 1°C. Based on our data (Si/Al anomaly 31, Mn/Fe anomaly 0.04, both ∼25% of the total observed range in μXRF data; Fig. 3, D and E), we suggest that a best maximum estimate lies around 0.5°C.

The combined data presented by this study demonstrate how detailed forensic analysis of multiple sedimentary proxies with sub-annual to multi-annual precision can provide robust empirical evidence of the environmental impacts of large volcanic eruptions, or indeed, other short-lived climate perturbations. In the case of the Toba eruption ∼74,000 years ago, our analysis of its registration in the high-quality record of Lake Chala indicates that, contrary to the long-standing high-impact paradigm (*9*–*11*), the largest volcanic event to have occurred during the existence of our species is unlikely to have significantly affected contemporaneous human populations in Africa. Our demonstration of modest impact is robust, firstly because the temporal resolution of our proxy data is high enough to capture the short-lived climate response to individual volcanic eruptions observed in model simulations (*12*, *13*), modern-day instrumental data (*57*) and less ancient climate-proxy records (*58*); and secondly because the limnological and ecological sensitivity of Lake Chala to regional climate variability is well established at both interannual (*33*, *34*) and longer time scales (*28*, *59*–*61*). In continental regions that experienced thick (re-) deposition of Toba ash, such as some locations in India (*62*, *63*), the Northern Hemisphere winter timing of the eruption may have affected the phenology of local vegetation and thus, potentially, the availability of food resources used by local humans. Exempting such areas, our results indicate that in (sub-) tropical regions at great distance from the Toba caldera, the Toba eruption caused no substantial climatic or environmental perturbation. Further, by eliminating one factor of uncertainty in eruption models, our constraint on the seasonal timing of the Toba eruption may improve model simulations of its global climatic and environmental impacts.

## MATERIALS AND METHODS

### Study site and materials

Lake Chala (3.19°S, 37.42°E; 879 m above sea level) is a volcanic crater lake in eastern equatorial Africa, straddling the Kenya-Tanzania border near Mt. Kilimanjaro. The ∼4.2 km^2^ lake has steep crater slopes all around its shoreline (Fig. 1B), continuing steeply underwater to 50–60 m depth and then more gradually to a depth of ∼90 m at the center (*60*). In the semi-arid tropical climate regime characterizing the Horn of Africa, lake-surface evaporation greatly exceeds mean annual precipitation, consequently long-term persistence of Lake Chala depends on substantial inflow of groundwater originating from rainfall on the upper forested slopes and subalpine zone of Mt. Kilimanjaro (*64*). The modern-day lake is permanently stratified (meromictic), i.e. annual mixing does not extend throughout the entire water column resulting in a turbulence-free and permanently anoxic profundal lake bottom. Deepest mixing (to 40–45 m depth) occurs during the main dry season in Southern Hemisphere winter (June–September) due to coincidence of lowest night-time air temperatures and increased windiness promoting deep convection (*33*). The water column is most strongly stratified during the two wet seasons, as well as during the intervening short dry season (January–February) in Southern Hemisphere summer because peak daytime air temperatures at that time of year counteract wind-driven turbulence by maintaining a sizable temperature (and thus density) contrast between surface and deeper water masses (∼27 and ∼22°C, respectively), preventing deep mixing (*34*, *36*).

Upcycling of dissolved nutrients from the lower water column during the main dry season stimulates a diatom bloom which is eventually registered in the sediment record as a light-colored lamina consisting mostly of diatom frustules. During the remainder of the year, a dark lamina consisting of diffuse organic and clay-sized mineral matter is deposited. When both light and dark laminae are consistently well-expressed, they from annual laminations or varves (*34*). The relative thickness of these varves mostly depends on variation in light lamina thickness, reflecting interannual climate variability that, at least today and in recorded history, is partly teleconnected to ENSO (*33*). Dry and slightly cooler conditions during La Niña phases promote deep dry-season mixing and thus more massive diatom blooms leading to thicker varves, whereas wet El Niño phases are associated with less deep seasonal mixing, weaker diatom blooms and thinner varves (*33*).

In 2016, the International Continental Scientific Drilling Programme (ICDP) project DeepCHALLA retrieved from Lake Chala a demonstrably continuous (*31*, *60*) lake-sediment sequence spanning the last ∼250 kyr (*30*). Five main core sequences (DCH-CHL16-1A to 1E) were drilled from adjacent holes in 3-m sections and compiled into a composite master sequence, which extends down to ∼214.8 m below the lake floor. Sieving and microscopic scanning of contiguous 0.5-cm core increments was carried out across a section that [based on age-model extrapolation; (*29*, *60*)] was estimated to contain the Toba time window. Cryptotephra DCH-67.55 (a macroscopically non-visible tephra) was detected at 45.5–45.0 cm in core section DCH-CHL16-1B-21H-2 (67.55 mcd), and geochemically confirmed as YTT ashfall originating from the 73.7 ± 0.3 kyr BP eruption of Mt. Toba, Indonesia (fig. S3) (*28*).

### Visualization of sediment structure

Fine-scale structure of the sediments bridging the YTT cryptotephra deposit was studied at 50–400× magnification under a polarizing microscope, on a thin section cut from a 10-cm-long resin-impregnated block of sediment covering the 39–49 cm interval in DCH-CHL16-1B-21H-2. Thin-section preparation was done at MKfactory Stahnsdorf (Germany), by shock-freezing the fresh sediment in liquid nitrogen followed by freeze-drying for 48 h and impregnation with Araldite resin (*65*).

X-ray micro-computed tomography (μCT) imaging of the YTT deposit and adjacent sediments was done on a 2 × 2 × 5-cm u-channel block of fresh sediment extracted from DCH-CHL16-1B-21H-2. Scans were executed with HECTOR, a high-energy μCT scanner at the Ghent University Centre for x-ray Tomography (UGCT) (*66*). The block was first scanned entirely by merging two overview scans at 13.1× magnification, using a tube voltage of 160 keV, tube power of 10 W and an aluminum filter of 1 mm thick. A total of 2400 x-ray images were captured with an exposure time of 2 s each. Then a region-of-interest (ROI) scan with 10 mm diameter and 32 mm height was produced by merging four individual scans obtained with identical settings as above but at 32.8× magnification. Volumes were reconstructed using Octopus Reconstruction 8.9.4.0 (*67*), resulting in voxel sizes of 15 and 6 μm for the overview and ROI scans, respectively. Visualization of the reconstructed volumes was done using VGStudio software version 2 (VolumeGraphics). Higher radiodensities are shown as brighter gray values. Movies S1 and S2 provide a 3D view of the scanned sediments around the YTT horizon.

### Sediment elemental compositions

Changes with depth in the elemental composition of the sediment were measured at 500-μm resolution by micro-x-ray fluorescence (μXRF) scanning along the center of the archival half of DCH-CHL16-1B-21H-2 using an Avaatech XRF Technology core scanner at the Max Planck Institute for Chemistry, Mainz (Germany). Operational settings of 10 kV tube voltage, 500 μA tube current, and 10 s acquisition time per spot were applied.

Changes in elemental concentrations were traced at μm-scale resolution via off-center linear scanning of the 40.5–50.4 cm interval, using an Element 2 inductively-coupled plasma mass spectrometer (ICP-MS) at the Max Planck Institute for Chemistry, Mainz (Germany) equipped with a high-energy Nd:YAG UP213 (213 nm wavelength) laser ablation system. Measurements were performed with a laser beam spot size of 80 μm, laser repetition rate of 10 Hz, and a scan speed of 10 μm s^−1^ resulting in an analytical resolution of ∼7 μm. The synthetic glass NIST SRM 612 was used as reference material for calibration (*68*), and pressed carbonate powder USGS MACS-3 for quality control (*69*). For all elements analyzed, measured values of the USGS MACS-3 standard were in agreement with GeoReM values (*70*).

### Stable carbon and nitrogen isotope analyses

For bulk-sediment analyses, 80 samples were extracted in contiguous 2–3-mm intervals from between ∼54.2 cm and ∼32.7 cm depth in DCH-CHL16-1B-21H-2 (Fig. 1C), corresponding to the period from ∼260 year before to ∼190 year after the Toba eruption based on triplicate varve counting (Supplementary Materials). Sample boundaries traced the depositional plane of macroscopically visible fine lamination in order to optimize comparison of bulk-composition data with mm-scale visual stratigraphy and high-resolution μXRF and ICP-MS data. Depletion of suitable material in the working half of DCH-CHL16-1B-21H-2 necessitated sampling the 53.2–46.0 cm interval in the equivalent interval (97.3–89.9 cm) of DCH-CHL16-1A-22H-3, with shared stratigraphy guaranteeing perfect temporal continuity (fig. S4).

Elemental concentrations of carbon and nitrogen and their stable isotopic signatures (δ^13^C and δ^15^N) in bulk organic matter were measured at the British Geological Survey (BGS). An aliquot of each sample was submerged in 5% hydrochloric acid overnight to remove any inorganic carbon (present in negligible amounts only), rinsed with deionized water, dried at 40°C, and homogenized to a fine powder using a pestle and mortar. The acid-rinsed samples were weighed in tin capsules using a microbalance to provide ∼0.4 mg C for C/N and δ^13^C analysis, and raw sediment was weighed to provide ∼0.1 mg N for δ^15^N analysis. The samples were combusted using an Elementar vario ISOTOPE cube connected online to an Elementar isoprime precisION isotope ratio mass spectrometer. Isotope values are reported in standard delta notation in per mil (‰) and normalized to the VPDB and AIR scales for δ^13^C and δ^15^N respectively, using a multi-point calibration based on the international reference materials USGS61 (−35.05‰ δ^13^C and − 2.87‰ δ^15^N), USGS62 (−14.79‰ δ^13^C and + 20.17‰ δ^15^N) and USGS63 (−1.17‰ δ^13^C and + 37.83‰ δ^15^N) (*71*), as well as internal reference BROC3 (−27.6‰ δ^13^C), all analyzed alongside the Lake Chala samples. External precision (1σ) for the international standards is 0.1‰ for δ^13^C and 0.2‰ for δ^15^N. BROC3 (41.25% C, 4.85% N) was used to calculate %C and %N values.

### Fossil diatom analysis

Lake Chala sediments generally contain abundant and well-preserved diatoms (fossil diatom frustule flux ∼10^10^ μm^3^ cm^−2^ year^−1^) (*42*), with fossil assemblages predominantly comprised of various *Nitzschia* species and *Afrocymbella barkeri* (*42*, *72*–*74*) (fig. S5). Consequently, weighed aliquots of the fresh mud samples extracted for bulk stable-isotope analysis could simply be diluted in deionized water and mounted onto glass slides for identification and counting of fossil diatoms under a polarizing microscope at 400× magnification. For robust quantification of percent composition, at least 300 fossil diatom valves were identified per sample.

## Supplementary Materials

### This PDF file includes:

Supplementary Text Figs. S1 to S5 Tables S1, S2, and S4 Legend for table S3 Legends for movies S1 and S2 References
