# Supplementary material for: Sub-annual resolution evidence for limited impact of the 74-ka Toba eruption on eastern African climate
Source: Sci Adv. 2026 Jul 31;12(31):eadv6851. doi: 10.1126/sciadv.adv6851 (PMC13440512; doi:10.1126/sciadv.adv6851)
Supplement: Supplementary file 1 [file sciadv.adv6851_sm.pdf]

Science Advances
AAAS

Supplementary Materials for

Sub-annual resolution evidence for limited impact of the 74-ka Toba eruption on eastern African climate

Jinheum Park *et al.*

Corresponding author: Jinheum Park, jinheum.park@uni-mainz.de

Sci. Adv. **12**, eadv6851 (2026)
DOI: 10.1126/sciadv.adv6851

The PDF file includes:

Supplementary Text
Figs. S1 to S5
Tables S1, S2, and S4
Legend for table S3
Legends for movies S1 and S2
References

Other Supplementary Material for this manuscript includes the following:

Movies S1 and S2
Table S3

Supplementary Text

Time period covered by this study

The duration of ~450 years assigned to the time period covered by this study is based on varve counting and must be considered approximative. Due to variation in white-lamina thickness the rate of sediment accumulation in Lake Chala can vary two- to five-fold on decade-to-century time scales (*33, 79*), as evidenced by the rapid alternation of light- and dark-colored sections (Fig. 1C) (*28*). Mostly well-expressed white laminae in the 12.7-cm section of DCH-CHL16-1B-21H-2 above YTT allowed varve counting with an error range of 5–7% based on triplicate counts, yielding average sedimentation rates of 0.70 mm/yr for the section of medium-dark muds immediately above YTT (54±3 varves) and 0.65 mm/yr for the light-brown colored upper half of the studied section (133±10 varves; Fig. 3H). Poor expression of white laminae in the analyzed 8.6-cm section of DCH-CHL16-1B-21H-2 below YTT prevented varve counting with sufficient accuracy to determine the duration of the studied pre-Toba period. Interpreting all faint white laminae in the 2.3-mm interval immediately below YTT (Fig. 2C) to represent dry-season diatom blooms yields an average sedimentation rate of 0.33 mm/yr. Assigning this rate to the entire section of similarly dark brown mud below YTT infers a duration of ~265 years for the studied pre-Toba period. Considering that the rate value of 0.33 mm/yr exceeds the average thickness of dark laminae recorded in 5,119 varves from within the last 25 kyr of the Lake Chala record (0.17±0.14 mm) (*33*), we surmise that ~265 years may under- rather than overestimate the true duration of the studied pre-Toba section of DCH-CHL16-1B-21H-2.

Possible effect of direct tephra input on the Lake Chala ecosystem

To test the possibility that Si-rich ashfall from the Toba eruption might have affected environmental conditions in Lake Chala directly through changes in aquatic geochemistry rather than climate forcing (*80-82*), we traced sedimentary proxy signals below and above the visible tephra DCH-67.56 (Fig. 1C, gray triangle), which originated from a volcanic eruption within the East African Rift System (*29*). No diatom bloom (preserved as a thick light varve lamina and locally elevated Si/Al ratio) comparable to the post-Toba diatom bloom is registered to have occurred following the deposition of DCH-67.56, notwithstanding that its momentary Si input to Lake Chala must have been several orders of magnitude higher than that due to YTT ashfall. Also, other proxies of aquatic productivity (Fig. 3, G to H) show little change across tephra DCH-67.56, logically so because Lake Chala is by nature a Si-rich environment (*42*). Therefore, total YTT-associated Si input from the even lesser amount of YTT ashfall (directly onto the lake surface and on the small area of steep-sloping crater walls; Fig. 1B) can be presumed negligible. Considering the small volume and very low P content of YTT glass shards (~0.02 wt % P_2O_3) (*29*), addition of P due to YTT ashfall can also be presumed trivial in relation to the nutrient budget available to phytoplankton. We thus surmise that the direct geochemical impact of tephra input to Lake Chala was minimal, and that ecosystem changes observed above the YTT ashfall deposit were mostly driven by climate forcing.

Derivation of Toba eruption season from the Lake Chala sediment sequence

Understanding the seasonality of sedimentation processes in Lake Chala is essential for accurate derivation of the time of year in which the Toba eruption occurred. The scenario presented in this study assumes no or limited discrepancy between the seasonal succession of different light- and dark-lamina varve components in the upper water column and the sequence in which these

components eventually settle on the lake bottom. With regard to the diatom frustules deposited in Lake Chala sediments, this assumption is supported by monthly monitoring data of the modern system, which show no or little temporal offset in the annual pattern of live diatom abundance within and immediately below the photic zone (respectively 0–10 m and 10–20 m depth) (*32*), and down to 35 m depth where a sediment trap captured dead diatoms sinking into the hypolimnion (*33*). Rapid sinking of diatom frustules from the photic zone is also demonstrated by their near-absence in the 0–10 m depth interval except during the June-September bloom season (*32*). Although no sediment trap data are available from greater depth, the lower water column is entirely free of water circulation that would slow down the descent of diatom frustules, as turbulent and convective mixing are limited to the upper ~20 m and ~45 m, respectively (*32, 33*). For reference, also in the ~60 m-deep fully mixing (holomictic) Lake Tiefer See, Germany (53.59° N, 12.53° E), little temporal offset is observed in diatom fluxes trapped at 12 and 50 m depth, implying rapid sinking of dead and dying diatoms after the annual bloom (*83*). Applying Stoke's law for laminar-flow conditions (*84*), diatoms in Lake Chala can be expected to reach the 88-m deep lake floor within 1–2 weeks, with larger *A. barkeri* frustules (20–64 µm long × 8–14 µm wide (*74, 85*); fig. S5A) sinking more rapidly than those of *Nitzschia* species (*N. fabiennejansseniana* 24–49 µm long × 2–3 µm wide (*75, 85*); fig. S5B). YTT glass shards (semi-rectangular with longest axis <130 µm) (*29*) likely descended through the entire water column in a few days (*84*). While clay-sized mineral and organic particles comprising each dark lamina must have settled more slowly, the near-purity of light diatom laminae indicates that delayed deposition of material formed or entering the lake during the previous wet season was minimal. Even if the delay effect was significant, the stratigraphic position of the Toba ashfall deposit relative to the bracketing light diatom laminae would not have been affected.

<u>Provenance</u>

Recovery of the Lake Chala sediment record was accomplished by the DeepCHALLA project, co-financed by the International Continental Scientific Drilling Programme (ICDP) and personal or institutional funding awarded to members of the DeepCHALLA science team. Permission was granted by the governments of Kenya and Tanzania through the National Commission for Science, Technology & Innovation (NACOSTI P/16/7890/10400), the National Environmental Management Authority (NEMA EIA/PSL/3851), the Commission for Science and Technology (COSTECH NA-2016-67.270-285 and NA-2016-201.277-292), the National Environmental Management Council (EIA/10/0143/V.I/04), and resident permits issued to all foreign-national scientists and engineers by the host countries' Departments of Immigration. Lake-drilling and project-staging operations were subject to environmental impact assessments conducted by Kamfor (Nairobi, Kenya) and Tansheq (Dar es Salaam, Tanzania), and permission from the Lands and Settlement Office of Taita-Taveta County (Kenya) for deploying research facilities on government land. Responsibility for long-term storage and curation of the DeepCHALLA core sequence, including sections DCH-CHL16-1B-21H-2 and DCH-CHL16-1A-22H-3 analyzed in this study, is shared by Ghent University (UGent) and the Flanders Marine Institute (VLIZ), using facilities partly financed by Hercules infrastructure grant AUGE/15/14-G0H2916N from the Research Foundation of Flanders (FWO). All derived research materials (sediment blocks extracted for CT-scanning, epoxy-embedded thin sections, freeze-dried sediments, etc.) are stored at UGent. Both sediment cores and derived materials can be accessed upon reasonable request to Dirk Verschuren (dirk.verschuren@UGent.be).

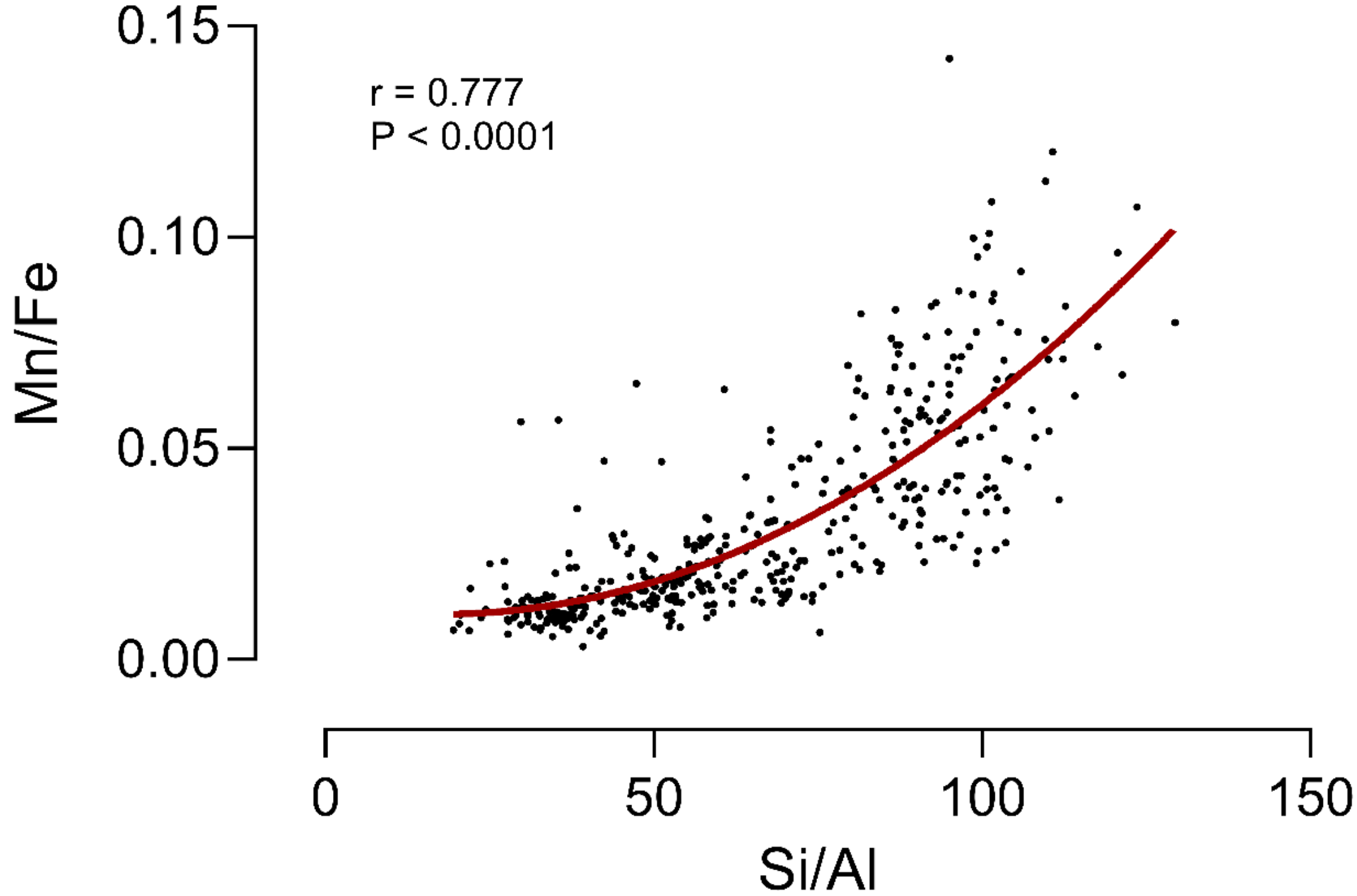

Fig. S1. Regression of Mn/Fe versus Si/Al in the 54.0-33.0 cm portion of core section DCH-CHL16-1B-21H-2 (73.78-73.57 mcd), based on µXRF scanning. The Pearson's correlation coefficient (r) of a 2nd-order polynomial least squares fit to the data (red line) is 0.777 ($P < 0.0001$; $n = 429$).

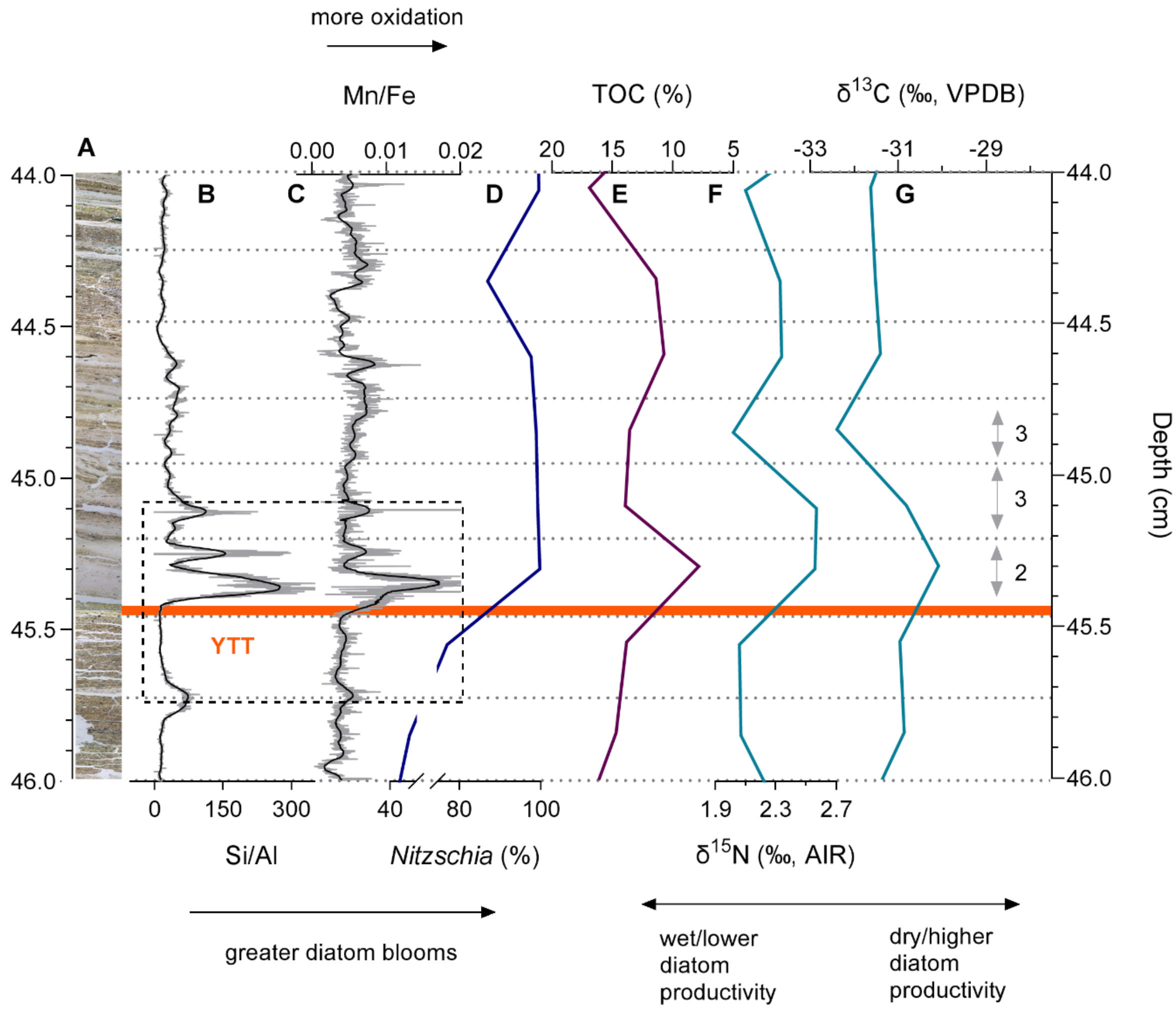

Fig. S2. Multi-proxy data for the portion of DCH-CHL16-1B-21H-2 between 46.0 and 44.0 cm (67.54–67.53 mcd), bracketing the YTT cryptotephra deposit at 45.4 cm (67.55 mcd). (**A**) Composite microscopic image of epoxy-embedded thin section. (**B**) Si/Al and (**C**) Mn/Fe, based on elemental abundances measured at ~7-µm resolution by LA-ICP-MS; the black lines are 100-point running means. Note the low Si/Al values at the stratigraphic position of the YTT glass-shard deposit (orange line), reflecting its very minor Si input compared with diatom and local mineral sources. (**D**) Percentage of *Nitzschia* specimens in the fossil diatom assemblage. (**E**) TOC; note the reversed scale. (**F**) $\delta^{15}N$ and (**G**) $\delta^{13}C$ of bulk organic matter. Stippled horizontal lines indicate the exact sample boundaries for the diatom, TOC and stable-isotopic analyses (data shown in D–G), with integer numbers at the right indicating the number of years represented by each sample as based on varve laminae counts. The rectangle across B and C delineates the ~6-mm-long section of the Si/Al and Mn/Fe profiles also shown in Fig. 2C.

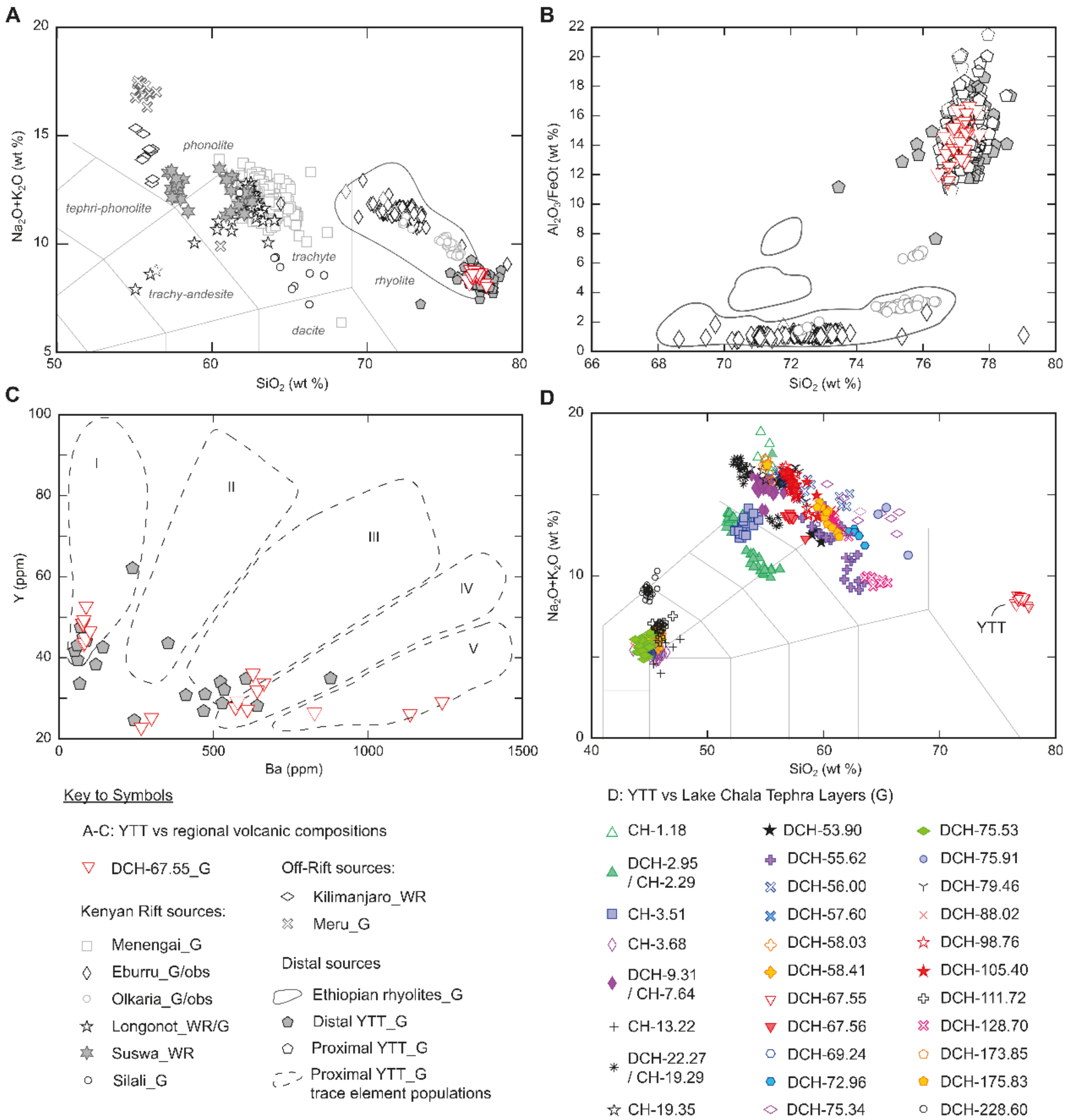

Fig. S3. Major, minor and trace element glass shard compositions of DCH-67.55 confirming the 73.7-ka YTT eruption as its source. (**A**) Total alali silica plot (*86*) compares major and minor element oxide compositions of glass shards in DCH-67.55 to evolved volcanic products (phonolitic, trachytic and rhyolitic only) from nearby volcanoes in Central Kenyan Rift (*87-95*) and Northern Tanzanian Off-Rift areas (*96, 97*), as well as distal Main Ethiopian Rift (*98*) volcanoes and YTT glass found in Lake Malawi (*23, 24, 99*). Where available, tephra glass shard compositions (G) are plotted for reference data, but these are supplemented by obsidian (obs) and whole rock (WR) compositions where data are limited. DCH-67.55 is rhyolitic and overlaps precisely with YTT glass compositions, the envelope of Main Ethiopian Rift compositions and close to the most evolved compositional data from Eburru and Olkaria (Central

Kenyan Rift). (**B**) takes those rhyolitic data and shows that DCH-67-55 is compositionally consistent with proximal (*100*) and distal (*23, 24, 99*) YTT glass compositions and distinct from any East African volcanic sources. (**C**) further confirms the DCH-67.55 correlation to YTT using trace element compositions. DCH-67.55 correlated to sub-populations I–IV of the YTT, recognized in samples from Malaysia and the Indian Subcontinent (*100*). (**D**) demonstrates the distinctiveness of DCH-67.55 compared to the glass shard major element compositions of 29 visible tephra layers geochemically characterized in the full DeepCHALLA sediment sequence (>250 kyr). Further information, and details of analytical methods, specific to DCH-67.55 is provided by ref. *28*. Glass compositional data for DCH-67.55 and all visible tephra layers are presented and discussed in ref. *29*.

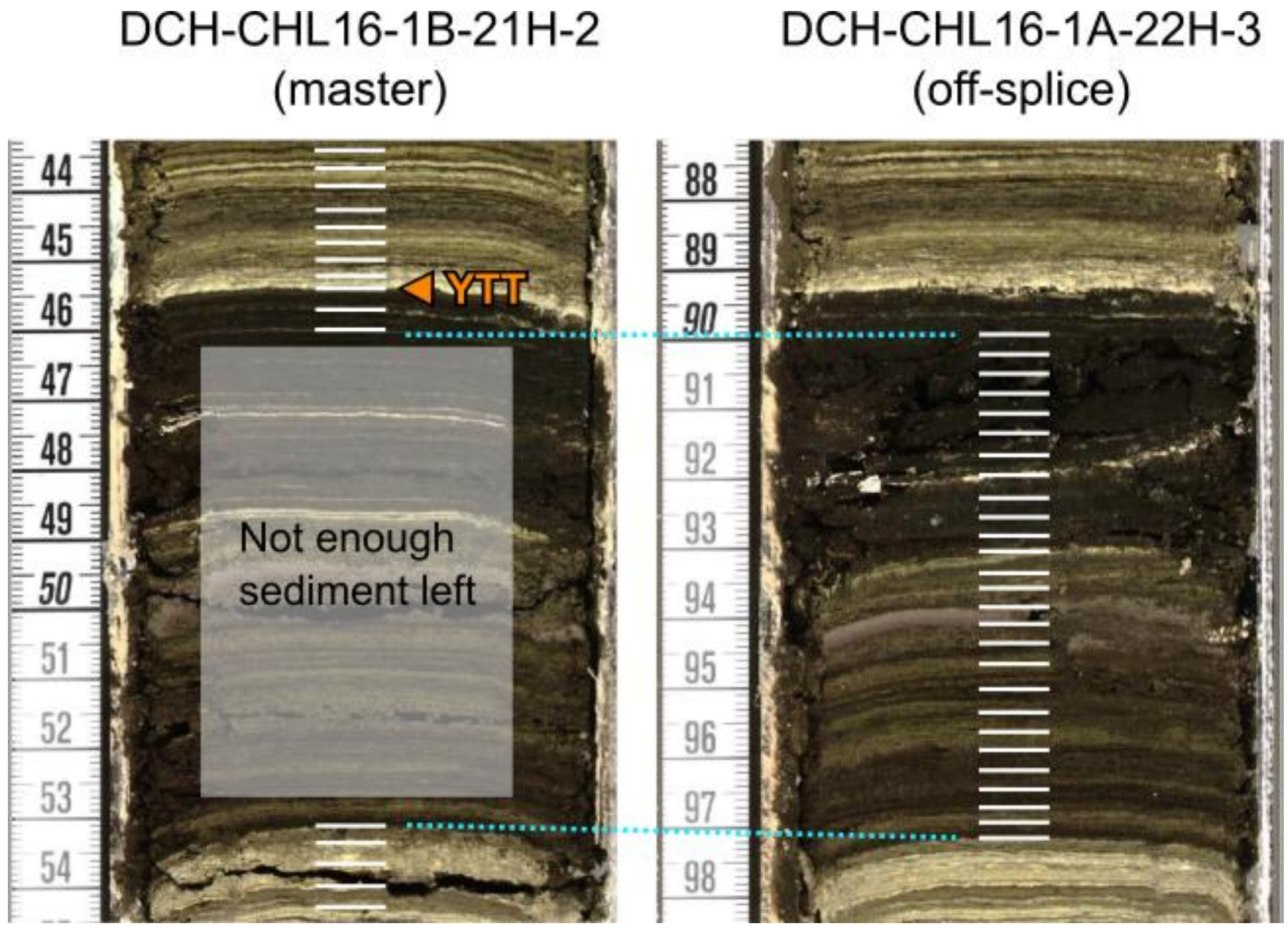

Fig. S4. Paired digital line-scan images of overlapping core sections DCH-CHL16-1B-21H-2 and -1A-22H-3, highlighting the perfect cross-correlation supporting contiguous close-interval sampling for bulk-sediment analyses. The white horizontal lines are positioned at the exact sampling boundaries. The cyan stippled lines denote the intervals where transition between the two cores was made, based on control points of the second diatom lamina below the YTT (top cyan stippled line) and the transition towards the light diatomaceous lamina-dominated interval (bottom cyan stippled line).

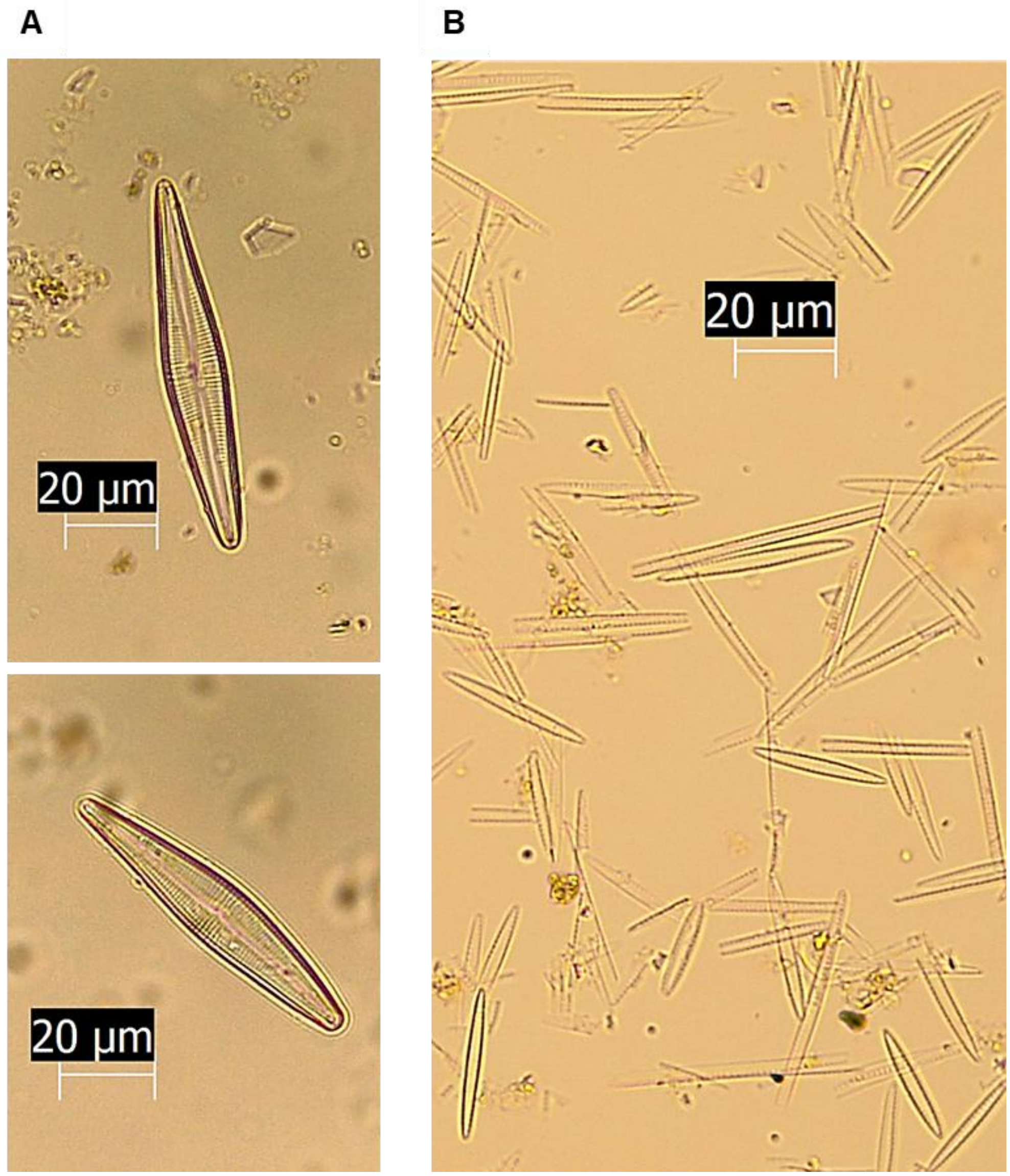

Fig. S5. Light-microscopic photos of fossil diatoms from Lake Chala sediments. (**A**) *Afrocymbella barkeri* (specimens from 92.3–92.6 cm depth in DCH-CHL-1A-22H-3). (**B**) *Nitzschia* spp. (specimens from 45.2–45.4 cm depth in DCH-CHL16-1B-21H-2). All photos taken at 400× magnification under a Leica ICC50 W microscope camera mounted on a Leica DM 750 polarizing microscope and displayed at identical scale.

Table S1. µXRF Si/Al and Mn/Fe values for sediments bracketing the YTT horizon in the DeepCHALLA sequence. The data are as shown in Fig. 3, D–E. The depth values refer to section depth in DCH-CHL16-1B-21H-2, aligned to the YTT horizon being fixed at exactly 45.4 cm.

Depth (cm)	Si/Al	Mn/Fe
33.15	112.690	0.084
33.20	96.370	0.055
33.25	102.309	0.066
33.30	121.354	0.067
33.35	100.792	0.043
33.40	65.990	0.016
33.45	71.237	0.026
33.50	88.162	0.033
33.55	94.006	0.057
33.60	104.108	0.047
33.65	96.825	0.072
33.70	98.066	0.074
33.75	123.642	0.107
33.80	108.063	0.053
33.85	120.697	0.096
33.90	107.640	0.059
33.95	129.412	0.080
34.00	111.707	0.038
34.05	100.645	0.035
34.10	90.403	0.027
34.15	79.201	0.033
34.20	81.773	0.027
34.25	88.384	0.038
34.30	94.706	0.059
34.35	110.257	0.054
34.40	114.176	0.062
34.45	110.757	0.120
34.50	101.464	0.108
34.55	92.237	0.084
34.60	100.742	0.040
34.65	90.450	0.032
34.70	95.018	0.065
34.75	104.047	0.066
34.80	86.829	0.083
34.85	93.639	0.057
34.90	90.586	0.035
34.95	86.189	0.076
34.95	86.102	0.064
34.95	110.097	0.071
35.00	99.153	0.078
35.05	102.808	0.080
35.10	103.321	0.071
35.15	99.313	0.095
35.20	101.836	0.087
35.25	95.061	0.142
35.30	98.728	0.100
35.35	90.461	0.058
35.40	103.648	0.028
35.45	82.155	0.062
35.50	86.383	0.051
35.55	91.607	0.062
35.60	81.168	0.067
35.65	67.822	0.052
35.70	60.014	0.026
35.75	58.220	0.018
35.80	56.464	0.022
35.85	57.896	0.028
35.90	67.915	0.033
35.95	60.940	0.017
36.00	58.616	0.029
36.05	63.968	0.026
36.10	73.658	0.048
36.15	76.072	0.043
36.20	80.368	0.039
36.25	61.021	0.029
36.30	68.610	0.016
36.35	81.372	0.021
36.40	99.413	0.026
36.45	87.796	0.031
36.50	78.327	0.026
36.55	80.940	0.050
36.60	96.249	0.043
36.65	88.491	0.052
36.70	80.944	0.064
36.75	89.430	0.070
36.80	81.607	0.082
36.85	89.744	0.038
36.90	103.711	0.035
36.95	96.575	0.051
37.00	94.623	0.041
37.05	80.429	0.057
37.10	89.586	0.042
37.15	88.932	0.041
37.20	90.634	0.059
37.25	84.722	0.022
37.30	84.436	0.038
37.35	101.900	0.041
37.40	95.060	0.063
37.45	92.982	0.084
37.50	87.459	0.074
37.55	92.272	0.065
37.60	103.804	0.060
37.65	89.038	0.056
37.70	95.535	0.055
37.75	87.159	0.059
37.80	85.992	0.063
37.85	94.695	0.042
37.90	77.347	0.032
37.95	94.175	0.029
38.00	96.198	0.040
38.05	106.990	0.046
38.10	101.113	0.101
38.15	87.242	0.072
38.20	97.418	0.052
38.25	90.889	0.035
38.30	99.141	0.023
38.35	93.288	0.054
38.40	102.342	0.038
38.45	80.517	0.036
38.50	87.177	0.041
38.55	94.792	0.069
38.60	91.308	0.058
38.65	104.597	0.067
38.70	100.349	0.059
38.75	101.952	0.064
38.80	101.774	0.055
38.85	91.561	0.077
38.90	91.170	0.023
38.95	86.466	0.047
39.00	112.175	0.076
39.05	105.534	0.078
39.10	100.787	0.098
39.15	112.351	0.071
39.20	85.346	0.054
39.25	95.626	0.027
39.30	74.282	0.015
39.35	69.407	0.013
39.40	61.912	0.013
39.45	58.211	0.013
39.50	62.123	0.019
39.55	65.892	0.030
39.60	79.641	0.041
39.65	78.826	0.040
39.70	88.374	0.056
39.75	105.954	0.092
39.80	98.578	0.086
39.85	117.627	0.074
39.90	103.558	0.048
39.95	101.519	0.085
39.95	96.486	0.087
39.95	95.654	0.072
40.00	91.983	0.056
40.05	97.566	0.035
40.10	86.888	0.075
40.15	109.615	0.076
40.20	99.674	0.039
40.25	93.100	0.029
40.30	96.642	0.030
40.35	78.403	0.047
40.40	88.691	0.064
40.45	77.122	0.025
40.50	48.723	0.016
40.55	41.910	0.006
40.60	44.808	0.015
40.65	90.372	0.038
40.70	109.725	0.113
40.75	96.831	0.044
40.80	102.134	0.026

40.85	93.877	0.040
40.90	88.099	0.042
40.95	91.376	0.040
41.00	83.951	0.023
41.05	80.575	0.023
41.10	74.129	0.014
41.15	70.500	0.015
41.20	67.769	0.020
41.25	75.440	0.035
41.30	83.804	0.040
41.35	86.759	0.069
41.40	88.834	0.063
41.45	88.115	0.054
41.50	89.455	0.041
41.55	70.546	0.016
41.60	65.299	0.014
41.65	69.484	0.016
41.70	66.472	0.014
41.75	75.719	0.017
41.80	72.270	0.023
41.85	57.147	0.028
41.90	50.098	0.024
41.95	37.082	0.025
42.00	35.452	0.057
42.05	47.813	0.017
42.10	53.563	0.020
42.15	38.211	0.022
42.20	45.191	0.011
42.25	59.808	0.020
42.30	72.471	0.048
42.35	86.415	0.034
42.40	72.926	0.033
42.45	70.071	0.022
42.50	68.238	0.019
42.55	70.951	0.019
42.60	70.053	0.014
42.65	67.199	0.023
42.70	69.479	0.021
42.75	67.974	0.025
42.80	63.815	0.026
42.85	67.822	0.038
42.90	70.408	0.032
42.95	76.666	0.030
43.00	71.771	0.022
43.05	73.010	0.023
43.10	68.741	0.024
43.15	60.167	0.023
43.20	53.111	0.017
43.25	45.464	0.017
43.30	52.180	0.015
43.35	52.171	0.019
43.40	52.298	0.019
43.45	49.453	0.012
43.50	57.189	0.027
43.55	55.082	0.029
43.60	54.181	0.020
43.65	56.182	0.026
43.70	57.482	0.016
43.75	60.900	0.027

43.80	63.751	0.031
43.85	57.685	0.028
43.90	55.175	0.027
43.95	51.339	0.020
44.00	44.201	0.011
44.05	49.409	0.014
44.10	52.697	0.009
44.15	53.391	0.014
44.20	52.847	0.011
44.25	61.995	0.016
44.30	58.046	0.010
44.35	52.357	0.008
44.40	55.720	0.015
44.45	57.290	0.018
44.50	59.142	0.013
44.55	59.625	0.022
44.60	64.587	0.034
44.65	75.751	0.039
44.70	67.339	0.032
44.75	69.749	0.019
44.80	58.361	0.023
44.85	52.825	0.019
44.90	50.226	0.017
44.95	48.323	0.021
44.95	54.195	0.018
44.95	55.431	0.023
45.00	64.781	0.034
45.05	58.371	0.033
45.10	58.368	0.029
45.15	68.425	0.033
45.20	64.079	0.043
45.25	63.819	0.021
45.30	58.128	0.010
45.35	42.427	0.007
45.40	36.987	0.007
45.45	37.338	0.011
45.50	44.043	0.013
45.55	44.727	0.014
45.60	46.313	0.015
45.65	42.080	0.019
45.70	41.514	0.017
45.75	41.971	0.010
45.80	39.294	0.011
45.85	37.906	0.010
45.90	30.838	0.011
45.95	27.851	0.014
46.00	30.838	0.010
46.05	34.540	0.012
46.10	38.992	0.010
46.15	38.828	0.015
46.20	38.910	0.013
46.25	37.384	0.013
46.30	34.400	0.010
46.35	34.617	0.005
46.40	39.261	0.003
46.45	41.274	0.009
46.50	39.551	0.017
46.55	41.925	0.014
46.60	39.581	0.012

46.65	36.231	0.009
46.70	35.132	0.011
46.75	33.148	0.014
46.80	37.261	0.012
46.85	35.419	0.011
46.90	30.884	0.009
46.95	27.800	0.006
47.00	27.915	0.009
47.05	30.532	0.009
47.10	28.943	0.010
47.15	22.054	0.017
47.20	25.033	0.023
47.25	32.454	0.011
47.30	35.051	0.010
47.35	32.344	0.013
47.40	29.819	0.012
47.45	31.351	0.014
47.50	40.353	0.007
47.55	33.767	0.015
47.60	34.149	0.011
47.65	34.639	0.015
47.70	36.239	0.011
47.75	39.178	0.015
47.80	39.202	0.013
47.85	37.927	0.012
47.90	36.867	0.010
47.95	34.094	0.012
48.00	33.580	0.015
48.05	32.637	0.010
48.10	35.482	0.009
48.15	37.765	0.010
48.20	33.814	0.009
48.25	32.777	0.008
48.30	33.675	0.009
48.35	35.662	0.011
48.40	36.127	0.010
48.45	37.905	0.010
48.50	36.541	0.011
48.55	36.717	0.009
48.60	37.780	0.011
48.65	36.335	0.009
48.70	29.821	0.008
48.75	29.883	0.012
48.80	32.210	0.013
48.85	34.964	0.011
48.90	30.812	0.015
48.95	30.105	0.013
49.00	28.975	0.012
49.05	29.299	0.012
49.10	35.046	0.009
49.15	34.605	0.013
49.20	36.133	0.008
49.25	31.859	0.008
49.30	32.723	0.007
49.35	27.743	0.010
49.40	21.877	0.007
49.45	20.386	0.009
49.50	19.499	0.007
49.55	20.576	0.011

49.60	23.689	0.010
49.65	24.392	0.012
49.70	28.710	0.010
49.75	29.912	0.014
49.80	32.355	0.014
49.85	33.654	0.014
49.90	37.170	0.022
49.95	27.398	0.017
49.95	27.311	0.023
49.95	35.013	0.020
50.00	46.362	0.013
50.05	40.527	0.015
50.10	40.774	0.015
50.15	49.638	0.025
50.20	56.972	0.017
50.25	56.321	0.027
50.30	56.023	0.021
50.35	44.346	0.027
50.40	46.616	0.026
50.45	38.389	0.036
50.50	43.680	0.029
50.55	49.718	0.019
50.60	49.433	0.019
50.65	48.413	0.013
50.70	51.316	0.016
50.75	51.004	0.019
50.80	48.346	0.017
50.85	50.455	0.015
50.90	49.789	0.014
50.95	56.860	0.018
51.00	53.562	0.014
51.05	63.283	0.020
51.10	54.082	0.019
51.15	50.195	0.018
51.20	54.825	0.020
51.25	53.383	0.015
51.30	49.835	0.015
51.35	53.007	0.015
51.40	46.097	0.025
51.45	43.841	0.029
51.50	29.748	0.056
51.55	45.508	0.030
51.60	46.449	0.020
51.65	52.535	0.014
51.70	54.252	0.014
51.75	49.945	0.018
51.80	52.029	0.010
51.85	50.310	0.013
51.90	54.052	0.008
51.95	54.331	0.008
52.00	59.068	0.011
52.05	54.531	0.014
52.10	55.207	0.020
52.15	53.041	0.018
52.20	58.779	0.016
52.25	53.465	0.020
52.30	52.906	0.015
52.35	48.133	0.016
52.40	45.344	0.014

52.45	37.358	0.014
52.50	37.130	0.013
52.55	38.203	0.013
52.60	44.962	0.014
52.65	43.396	0.019
52.70	45.622	0.015
52.75	44.193	0.011
52.80	51.788	0.018
52.85	53.322	0.019
52.90	56.979	0.019
52.95	47.062	0.013
53.00	48.639	0.020
53.05	53.908	0.021
53.10	58.007	0.034
53.15	68.799	0.021
53.20	72.891	0.015
53.25	64.827	0.027
53.30	78.367	0.020
53.35	71.047	0.046
53.40	75.158	0.051
53.45	81.954	0.044
53.50	99.675	0.053
53.55	79.591	0.070
53.60	94.857	0.078
53.65	96.440	0.069
53.70	67.855	0.054
53.75	42.413	0.047
53.80	47.365	0.065
53.85	51.208	0.047
53.90	60.797	0.064
53.95	71.523	0.041
54.00	83.445	0.041
54.05	80.522	0.029
54.10	84.389	0.021
54.15	75.350	0.006

Table S2. Bulk organic carbon (TOC) and nitrogen (N) content, C/N ratio, and carbon and nitrogen stable-isotope values for sediments bracketing the YTT horizon in the DeepCHALLA sequence. The data are as shown in Fig. 3, F–H and fig. S2. Depth values as in Table S1.

Top depth (cm)	Bottom depth (cm)	Medium depth (cm)	TOC %	N %	C/N	$\delta^{13}C$ ‰ VPDB	$\delta^{15}N$ ‰ AIR
32.70	33.00	32.85	3.98	0.16	24.6	-30.68	2.38
33.00	33.20	33.10	3.41	0.17	20.5	-30.83	2.60
33.20	33.40	33.30	5.77	0.20	28.8	-31.47	1.81
33.40	33.70	33.55	8.93	0.29	31.3	-31.78	2.14
33.70	34.00	33.85	5.39	0.20	26.7	-30.50	2.12
34.00	34.30	34.15	4.58	0.14	32.3	-31.27	2.29
34.30	34.50	34.40	7.98	0.24	33.5	-30.86	2.20
34.50	34.70	34.60	3.92	0.15	25.4	-30.72	2.69
34.70	35.00	34.85	5.60	0.17	32.5	-30.82	2.42
35.00	35.30	35.15	6.41	0.19	33.9	-31.58	2.26
35.30	35.50	35.40	4.06	0.14	29.6	-30.26	2.75
35.50	35.80	35.65	6.59	0.21	31.2	-30.75	2.25
35.80	36.10	35.95	10.10	0.39	26.0	-32.37	2.53
36.10	36.40	36.25	8.22	0.26	31.4	-31.23	2.44
36.40	36.70	36.55	7.31	0.24	30.8	-30.96	2.48
36.70	37.00	36.85	5.86	0.19	30.9	-30.82	2.67
37.00	37.20	37.10	7.23	0.21	34.5	-31.58	2.56
37.20	37.50	37.35	5.82	0.18	32.1	-30.56	2.68
37.50	37.80	37.65	6.86	0.23	29.3	-31.06	2.32
37.80	38.00	37.90	6.80	0.23	29.9	-30.72	2.13
38.00	38.30	38.15	6.62	0.23	29.2	-31.68	2.22
38.30	38.60	38.45	9.40	0.30	31.1	-30.87	2.30
38.60	38.90	38.75	7.22	0.21	33.9	-32.23	2.21
38.90	39.20	39.05	7.46	0.23	32.3	-31.28	2.41
39.20	39.50	39.35	8.34	0.27	30.8	-30.38	2.40
39.50	39.80	39.65	5.94	0.20	29.8	-29.61	2.49
39.80	40.10	39.95	4.79	0.15	31.8	-30.08	2.82
40.10	40.40	40.25	4.24	0.14	29.4	-30.93	2.76
40.40	40.60	40.50	5.77	0.19	29.6	-31.02	2.72
40.60	40.80	40.70	4.82	0.17	28.4	-29.53	2.88
40.80	41.00	40.90	4.34	0.15	29.6	-30.70	2.95
41.00	41.30	41.15	7.51	0.24	31.3	-30.91	2.82
41.30	41.60	41.45	5.97	0.21	28.3	-30.64	3.01
41.60	41.90	41.75	7.87	0.26	30.1	-31.00	2.61
41.90	42.10	42.00	7.63	0.26	29.0	-31.07	2.77
42.10	42.40	42.25	8.91	0.32	28.1	-30.98	3.21
42.40	42.60	42.50	9.29	0.32	28.9	-30.88	2.97
42.60	42.90	42.75	9.35	0.34	27.4	-31.41	3.11
42.90	43.20	43.05	10.58	0.38	28.1	-31.07	2.87
43.20	43.40	43.30	12.57	0.43	29.4	-31.00	3.18
43.40	43.70	43.55	12.39	0.42	29.4	-30.99	3.03
43.70	43.90	43.80	10.69	0.37	28.6	-30.98	2.81
43.90	44.20	44.05	16.87	0.52	32.7	-31.62	2.10
44.20	44.50	44.35	11.36	0.32	35.9	-31.52	2.33
44.50	44.70	44.60	10.71	0.32	33.9	-31.40	2.34

44.70	45.00	44.85	13.55	0.43	31.6	-32.39	2.02
45.00	45.20	45.10	13.90	0.43	32.2	-30.81	2.57
45.20	45.40	45.30	7.83	0.28	28.1	-30.08	2.56
45.40	45.70	45.55	13.81	0.49	28.4	-30.96	2.06
45.70	46.00	45.85	14.67	0.51	28.6	-30.86	2.07
46.00	46.17	46.09	16.86	0.59	28.7	-31.62	2.31
46.17	46.34	46.26	16.73	0.60	28.1	-31.31	2.57
46.34	46.51	46.43	16.23	0.59	27.4	-31.54	2.28
46.51	46.69	46.60	15.39	0.52	29.7	-32.89	2.20
46.69	46.86	46.77	13.89	0.45	30.7	-30.04	1.80
46.86	47.03	46.94	14.84	0.51	29.3	-29.69	2.06
47.03	47.20	47.11	14.18	0.47	29.9	-30.73	1.86
47.20	47.58	47.39	14.05	0.45	31.4	-29.38	2.05
47.58	47.96	47.77	14.55	0.49	29.9	-29.80	1.83
47.96	48.22	48.09	14.93	0.48	31.0	-31.22	1.85
48.22	48.60	48.41	16.69	0.47	35.8	-33.00	2.26
48.60	48.90	48.75	18.19	0.41	44.1	-32.36	1.67
48.90	49.20	49.05	14.44	0.47	30.6	-34.06	1.90
49.20	49.40	49.30	7.82	0.22	35.8	-30.29	2.46
49.40	49.62	49.51	9.59	0.27	35.3	-30.66	2.54
49.62	49.96	49.79	6.32	0.18	34.2	-28.22	2.42
49.96	50.29	50.13	11.89	0.32	37.4	-31.71	2.03
50.29	50.63	50.46	16.63	0.44	38.0	-34.11	2.41
50.63	50.96	50.80	16.56	0.42	39.0	-34.06	2.49
50.96	51.30	51.13	14.57	0.42	34.8	-33.95	2.48
51.30	51.64	51.47	13.72	0.37	37.2	-33.16	2.16
51.64	51.97	51.80	14.16	0.41	34.7	-31.76	2.61
51.97	52.31	52.14	15.59	0.44	35.3	-32.14	2.23
52.31	52.64	52.47	19.62	0.57	34.3	-33.55	2.10
52.64	52.98	52.81	12.77	0.34	37.2	-30.82	2.19
52.98	53.20	53.09	14.35	0.39	36.5	-32.05	1.90
53.20	53.40	53.30	10.28	0.28	36.1	-29.76	2.26
53.40	53.70	53.55	6.97	0.19	35.9	-32.23	2.01
53.70	54.00	53.85	7.56	0.22	34.8	-32.04	1.93
54.00	54.20	54.10	9.62	0.26	36.3	-31.68	2.03

Table S3 (separate file). LA-ICP-MS Si/Al and Mn/Fe values for sediments in the 44-46 cm portion of DCH-CHL16-1B-21H-2. The data are as shown in Fig. 2C and fig. S2, B and C.

Table S4. Counts of fossil diatoms in eight contiguous 0.2-cm or 0.3-cm increments spanning the 43.9 and 46.0 cm portion of DCH-CHL16-1B-21H-2, and percent *Nitzschia* in the total diatom assemblage. The data are as shown in fig. S2D.

Top depth (cm)	Bottom depth (cm)	*Nitzschia* counts	Total diatom counts	*Nitzschia* (% total diatoms)
43.9	44.2	441	443	99.55
44.2	44.5	389	447	87.02
44.5	44.7	456	467	97.64
44.7	45.0	618	625	98.88
45.0	45.2	423	426	99.30
45.2	45.4	404	405	99.75
45.4	45.7	614	796	77.14
45.7	46.0	251	452	55.53

Movie S1 (separate file). Animation cutting through an overview µCT scan of the U-channel sediment block (20 mm inner width, 50 mm high) containing the Lake Chala YTT deposit in its lower half.

Movie S2 (separate file). Animation circling around a region-of-interest µCT scan of the U-channel sediment block (10 mm diameter, 32 mm high) centered on the Lake Chala YTT deposit. Compare with Fig. 2B.

REFERENCES

1. D. F. Mark, P. R. Renne, R. C. Dymock, V. C. Smith, J. I. Simon, L. E. Morgan, R. A. Staff, B. S. Ellis, N. J. Pearce, High-precision $^{40}Ar/^{39}Ar$ dating of Pleistocene tuffs and temporal anchoring of the Matuyama-Brunhes boundary. *Quat. Geochronol.* **39**, 1–23 (2017).

2. D. Ninkovich, R. Sparks, M. Ledbetter, The exceptional magnitude and intensity of the Toba eruption, Sumatra: an example of the use of deep-sea tephra layers as a geological tool. *Bull. Volcanol.* **41**, 286–298 (1978).

3. W. I. Rose, C. A. Chesner, Dispersal of ash in the great Toba eruption, 75 ka. *Geology* **15**, 913–917 (1987).

4. A. Costa, V. C. Smith, G. Macedonio, N. E. Matthews, The magnitude and impact of the Youngest Toba Tuff super-eruption. *Front. Earth Sci.* **2**, 16 (2014).

5. M. G. Wiesner, A. Wetzel, S. G. Catane, E. L. Listanco, H. T. Mirabueno, Grain size, areal thickness distribution and controls on sedimentation of the 1991 Mount Pinatubo tephra layer in the South China Sea. *Bull. Volcanol.* **66**, 226–242 (2004).

6. M. R. Rampino, S. Self, Volcanic winter and accelerated glaciation following the Toba super-eruption. *Nature* **359**, 50–52 (1992).

7. G. A. Zielinski, P. A. Mayewski, L. D. Meeker, S. Whitlow, M. S. Twickler, K. Taylor, Potential atmospheric impact of the Toba Mega-Eruption∼ 71,000 years ago. *Geophys. Res. Lett.* **23**, 837–840 (1996).

8. J. Lin, P. M. Abbott, M. Sigl, J. P. Steffensen, R. Mulvaney, M. Severi, A. Svensson, Bipolar ice-core records constrain possible dates and global radiative forcing following the ∼74 ka Toba eruption. *Quat. Sci. Rev.* **312**, 108162 (2023).

9. M. R. Rampino, S. H. Ambrose, Volcanic winter in the Garden of Eden: the Toba supereruption and the late Pleistocene human population crash. *Spec. Pap. Geol. Soc. Am.* **345**, 71–82 (2000).

10. S. H. Ambrose, Late Pleistocene human population bottlenecks, volcanic winter, and differentiation of modern humans. *J. Hum. Evol.* **34**, 623–651 (1998).

11. J. Kappelman, L. C. Todd, C. A. Davis, T. E. Cerling, M. Feseha, A. Getahun, R. Johnsen, M. Kay, G. A. Kocurek, B. A. Nachman, A. Negash, T. Negash, K. O'Brien, M. Pante, M. Ren, E. I. Smith, N. J. Tabor, D. Tewabe, H. Wang, D. Yang, S. Yirga, J. W. Crowell, M. F. Fanuka, T. Habtie, J. N. Hirniak, C. Klehm, N. D. Loewen, S. Melaku, S. M. Melton, T. S. Myers, S. Millonig, M. C. Plummer, K. J. Riordan, N. A. Rosenau, A. Skinner, A. K. Thompson, L. M. Trombette, A. Witzel, E. Assefa, M. Bodansky, A. A. Desta, C. J. Campisano, D. Dalmas, C. Elliott, M. Endalamaw, N. J. Ford, F. Foster, T. Getachew, Y. L. Haney, B. H. Ingram, J. Jackson, C. W. Marean, S. Mattox, K. de la Cruz, Medina, G. Mulubrhan, K. Porter, A. Roberts, P. Santillan, A. Sollenberger, J. Sponholtz, J. Valdes, L. Wyman, M. Yadeta, S. Yanny, Adaptive foraging behaviours in the Horn of Africa during Toba supereruption. *Nature* **628**, 365–372 (2024).

12. B. A. Black, J.-F. Lamarque, D. R. Marsh, A. Schmidt, C. G. Bardeen, Global climate disruption and regional climate shelters after the Toba supereruption. *Proc. Natl. Acad. Sci. U.S.A.* **118**, e2013046118 (2021).

13. C. Timmreck, H. F. Graf, S. J. Lorenz, U. Niemeier, D. Zanchettin, D. Matei, J. H. Jungclaus, T. J. Crowley, Aerosol size confines climate response to volcanic super-eruptions. *Geophys. Res. Lett.* **37**, L24705 (2010).

14. A. Robock, C. M. Ammann, L. Oman, D. Shindell, S. Levis, G. Stenchikov, Did the Toba volcanic eruption of ∼74 ka BP produce widespread glaciation? *J. Geophys. Res. Atmos.* **114**, (2009).

15. C. Oppenheimer, Limited global change due to the largest known Quaternary eruption, Toba ≈74 kyr BP? *Quat. Sci. Rev.* **21**, 1593–1609 (2002).

16. M. Khodri, T. Izumo, J. Vialard, S. Janicot, C. Cassou, M. Lengaigne, J. Mignot, G. Gastineau, E. Guilyardi, N. Lebas, A. Robock, M. J. McPhaden, Tropical explosive volcanic eruptions can trigger El Niño by cooling tropical Africa. *Nat. Commun.* **8**, 778 (2017).

17. S. Stevenson, J. T. Fasullo, B. L. Otto-Bliesner, R. A. Tomas, C. Gao, Role of eruption season in reconciling model and proxy responses to tropical volcanism. *Proc. Natl. Acad. Sci. U.S.A.* **114**, 1822–1826 (2017).

18. S. van der Kaars, M. A. Williams, F. Bassinot, F. Guichard, E. Moreno, F. Dewilde, E. J. Cook, The influence of the ∼73 ka Toba super-eruption on the ecosystems of northern Sumatra as recorded in marine core BAR94-25. *Quat. Int.* **258**, 45–53 (2012).

19. C. Y. Huang, M. Zhao, C. C. Wang, G. Wei, Cooling of the South China Sea by the Toba eruption and correlation with other climate proxies ∼71,000 years ago. *Geophys. Res. Lett.* **28**, 3915–3918 (2001).

20. M. A. Williams, S. H. Ambrose, S. van der Kaars, C. Ruehlemann, U. Chattopadhyaya, J. Pal, P. R. Chauhan, Environmental impact of the 73 ka Toba super-eruption in South Asia. *Palaeogeogr. Palaeoclimatol. Palaeoecol.* **284**, 295–314 (2009).

21. H. Schulz, K.-C. Emeis, H. Erlenkeuser, U. von Rad, C. Rolf, The Toba volcanic event and interstadial/stadial climates at the marine isotopic stage 5 to 4 transition in the northern Indian Ocean. *Quatern. Res.* **57**, 22–31 (2002).

22. M. Haslam, C. Clarkson, M. Petraglia, R. Korisettar, S. Jones, C. Shipton, P. Ditchfield, S. H. Ambrose, The 74 ka Toba super-eruption and southern Indian hominins: archaeology, lithic technology and environments at Jwalapuram Locality 3. *J. Archaeol. Sci.* **37**, 3370–3384 (2010).

23. E. I. Smith, Z. Jacobs, R. Johnsen, M. Ren, E. C. Fisher, S. Oestmo, J. Wilkins, J. A. Harris, P. Karkanas, S. Fitch, A. Ciravolo, D. Keenan, N. Cleghorn, C. S. Lane, T. Matthews, C. W. Marean, Humans thrived in South Africa through the Toba eruption about 74,000 years ago. *Nature* **555**, 511–515 (2018).

24. C. S. Lane, B. T. Chorn, T. C. Johnson, Ash from the Toba supereruption in Lake Malawi shows no volcanic winter in East Africa at 75 ka. *Proc. Natl. Acad. Sci. U.S.A.* **110**, 8025–8029 (2013).

25. L. J. Jackson, J. R. Stone, A. S. Cohen, C. L. Yost, High-resolution paleoecological records from Lake Malawi show no significant cooling associated with the Mount Toba supereruption at ca. 75 ka. *Geology* **43**, 823–826 (2015).

26. C. L. Yost, L. J. Jackson, J. R. Stone, A. S. Cohen, Subdecadal phytolith and charcoal records from Lake Malawi, East Africa imply minimal effects on human evolution from the ∼74 ka Toba supereruption. *J. Hum. Evol.* **116**, 75–94 (2018).

27. J. D. Guballa, J. Bollmann, K. Schmidt, A. Lückge, The Toba eruption 74,000 years ago strengthened the Indian winter monsoon-evidence from coccolithophores. *Paleoceanogr. Paleoclimatol.* **39**, e2023PA004823 (2024).

28. A. Baxter, D. Verschuren, F. Peterse, D. Miralles, C. Martin-Jones, A. Maitituerdi, T. van der Meeren, M. van Daele, C. Lane, G. Haug, D. O. Olago, J. S. Sinninghe Damsté, Reversed Holocene temperature–moisture relationship in the Horn of Africa. *Nature* **620**, 336–343 (2023).

29. C. Martin-Jones, C. S. Lane, M. Blaauw, D. F. Mark, D. Verschuren, T. van der Meeren, M. van Daele, H. Wynton, N. Blegen, M. Kisaka, M. J. Leng, P. Barker, The >250-kyr Lake Chala record: A tephrostratotype correlating archaeological, palaeoenvironmental and volcanic sequences across eastern Africa. *Quat. Sci. Rev.* **326**, 108476 (2024).

30. D. Verschuren, D. O. Olago, S. M. Rucina, P. O. Odhengo, DeepCHALLA: Two glacial cycles of climate and ecosystem dynamics from equatorial East Africa. *Sci. Drill.* **15**, 72–76 (2013).

31. A. Maitituerdi, M. Van Daele, D. Verschuren, M. De Batist, N. Waldmann, Depositional history of Lake Chala (Mt. Kilimanjaro, equatorial East Africa) from high-resolution seismic stratigraphy. *J. Afr. Earth Sci.* **189**, 104499 (2022).

32. L. van Bree, F. Peterse, M. Van der Meer, J. Middelburg, A. Negash, W. De Crop, C. Cocquyt, J. Wieringa, D. Verschuren, J. S. Damsté, Seasonal variability in the abundance and stable carbon-isotopic composition of lipid biomarkers in suspended particulate matter from

a stratified equatorial lake (Lake Chala, Kenya/Tanzania): Implications for the sedimentary record. *Quat. Sci. Rev.* **192**, 208–224 (2018).

33. C. Wolff, G. H. Haug, A. Timmermann, J. S. S. Damsté, A. Brauer, D. M. Sigman, M. A. Cane, D. Verschuren, Reduced interannual rainfall variability in East Africa during the last ice age. *Science* **333**, 743–747 (2011).

34. C. Wolff, I. Kristen-Jenny, G. Schettler, B. Plessen, H. Meyer, P. Dulski, R. Naumann, A. Brauer, D. Verschuren, G. H. Haug, Modern seasonality in Lake Challa (Kenya/Tanzania) and its sedimentary documentation in recent lake sediments. *Limnol. Oceanogr.* **59**, 1621–1636 (2014).

35. W. De Crop, D. Verschuren, Determining patterns of stratification and mixing in tropical crater lakes through intermittent water-column profiling: A case study in western Uganda. *J. Afr. Earth Sci.* **153**, 17–30 (2019).

36. L. K. Buckles, J. W. Weijers, D. Verschuren, J. S. S. Damsté, Sources of core and intact branched tetraether membrane lipids in the lacustrine environment: Anatomy of Lake Challa and its catchment, equatorial East Africa. *Geochim. Cosmochim. Acta* **140**, 106–126 (2014).

37. K. D. Hoagland, J. R. Rosowski, M. R. Gretz, S. C. Roemer, Diatom extracellular polymeric substances: function, fine structure, chemistry, and physiology. *J. Phycol.* **29**, 537–566 (1993).

38. M. Toohey, K. Krüger, U. Niemeier, C. Timmreck, The influence of eruption season on the global aerosol evolution and radiative impact of tropical volcanic eruptions. *Atmos. Chem. Phys.* **11**, 12351–12367 (2011).

39. S. Osipov, G. Stenchikov, K. Tsigaridis, A. N. LeGrande, S. E. Bauer, M. Fnais, J. Lelieveld, The Toba supervolcano eruption caused severe tropical stratospheric ozone depletion. *Commun. Earth Environ.* **2**, 71 (2021).

40. M. P. McCormick, L. W. Thomason, C. R. Trepte, Atmospheric effects of the Mt Pinatubo eruption. *Nature* **373**, 399–404 (1995).

41. J. E. Kutzbach, Monsoon climate of the early Holocene: climate experiment with the earth's orbital parameters for 9000 years ago. *Science* **214**, 59–61 (1981).

42. P. A. Barker, E. R. Hurrell, M. J. Leng, B. Plessen, C. Wolff, D. J. Conley, E. Keppens, I. Milne, B. F. Cumming, K. R. Laird, C. P. Kendrick, P. M. Wynn, D. Verschuren, Carbon cycling within an East African lake revealed by the carbon isotope composition of diatom silica: a 25-ka record from Lake Challa, Mt. Kilimanjaro. *Quat. Sci. Rev.* **66**, 55–63 (2013).

43. E. R. Hurrell, P. A. Barker, M. J. Leng, C. H. Vane, P. Wynn, C. P. Kendrick, D. Verschuren, F. Alayne Street-Perrott, Developing a methodology for carbon isotope analysis of lacustrine diatoms. *Rapid Commun. Mass Spectrom.* **25**, 1567–1574 (2011).

44. M. Stoffel, M. Khodri, C. Corona, S. Guillet, V. Poulain, S. Bekki, J. Guiot, B. H. Luckman, C. Oppenheimer, N. Lebas, Estimates of volcanic-induced cooling in the Northern Hemisphere over the past 1,500 years. *Nat. Geosci.* **8**, 784–788 (2015).

45. C. Bühring, M. Sarnthein, L. Party, Toba ash layers in the South China Sea: evidence of contrasting wind directions during eruption ca. 74 ka. *Geology* **28**, 275–278 (2000).

46. C. Timmreck, Modeling the climatic effects of large explosive volcanic eruptions. *Wiley Interdiscip. Rev.-Clim. Chang.* **3**, 545–564 (2012).

47. L. Crick, A. Burke, W. Hutchison, M. Kohno, K. A. Moore, J. Savarino, E. A. Doyle, S. Mahony, S. Kipfstuhl, J. W. Rae, R. C. Steele, S. J. Sparks, E. W. Wolff, New insights into the ~74 ka Toba eruption from sulfur isotopes of polar ice cores. *Clim. Past* **17**, 2119–2137 (2021).

48. S. E. Nicholson, The nature of rainfall variability over Africa on time scales of decades to millenia. *Glob. Planet. Change* **26**, 137–158 (2000).

49. A. Svensson, M. Bigler, T. Blunier, H. B. Clausen, D. Dahl-Jensen, H. Fischer, S. Fujita, K. Goto-Azuma, S. Johnsen, K. Kawamura, S. Kipfstuhl, M. Kohno, F. Parrenin, T. Popp, S. O. Rasmussen, J. Schwander, I. K. Seierstad, M. Severi, J. P. Steffensen, R. Udisti, R. Uemura,

P. Vallelonga, B. Vinther, A. Wegner, F. Wilhelms, M. Winstrup, Direct linking of Greenland and Antarctic ice cores at the Toba eruption (74 ka BP). *Clim. Past* **9**, 749–766 (2013).

50. North Greenland Ice Core Project members, High-resolution record of Northern Hemisphere climate extending into the last interglacial period. *Nature* **431**, 147–151 (2004).

51. W. Du, H. Cheng, Y. Xu, X. Yang, P. Zhang, L. Sha, H. Li, X. Zhu, M. Zhang, N. M. Strikis, F. W. Cruz, E. Lawrence, H. Zhang, Y. Ning, Timing and structure of the weak Asian Monsoon event about 73,000 years ago. *Quat. Geochronol.* **53**, 101003 (2019).

52. A. Ganopolski, S. Rahmstorf, Rapid changes of glacial climate simulated in a coupled climate model. *Nature* **409**, 153–158 (2001).

53. Y. Wang, H. Cheng, R. L. Edwards, X. Kong, X. Shao, S. Chen, J. Wu, X. Jiang, X. Wang, Z. An, Millennial-and orbital-scale changes in the East Asian monsoon over the past 224,000 years. *Nature* **451**, 1090–1093 (2008).

54. K. A. Hughen, T. J. Heaton, Updated Cariaco Basin 14C calibration dataset from 0–60 cal kyr BP. *Radiocarbon* **62**, 1001–1043 (2020).

55. G. Deplazes, A. Lückge, L. C. Peterson, A. Timmermann, Y. Hamann, K. A. Hughen, U. Röhl, C. Laj, M. A. Cane, D. M. Sigman, G. H. Haug, Links between tropical rainfall and North Atlantic climate during the last glacial period. *Nat. Geosci.* **6**, 213–217 (2013).

56. S. E. Loomis, J. M. Russell, D. Verschuren, C. Morrill, G. De Cort, J. S. Sinninghe Damsté, D. Olago, H. Eggermont, F. A. Street-Perrott, M. A. Kelly, The tropical lapse rate steepened during the Last Glacial Maximum. *Sci. Adv.* **3**, e1600815 (2017).

57. D. H. Douglass, R. S. Knox, Climate forcing by the volcanic eruption of Mount Pinatubo. *Geophys. Res. Lett.* **32**, L05710 (2005).

58. J. Esper, L. Schneider, P. J. Krusic, J. Luterbacher, U. Büntgen, M. Timonen, F. Sirocko, E. Zorita, European summer temperature response to annually dated volcanic eruptions over the past nine centuries. *Bull. Volcanol.* **75**, 736 (2013).

59. D. Verschuren, J. S. Sinninghe Damsté, J. Moernaut, I. Kristen, M. Blaauw, M. Fagot, G. H. Haug, CHALLACEA project members, Half-precessional dynamics of monsoon rainfall near the East African Equator. *Nature* **462**, 637–641 (2009).

60. J. Moernaut, D. Verschuren, F. Charlet, I. Kristen, M. Fagot, M. De Batist, The seismic-stratigraphic record of lake-level fluctuations in Lake Challa: Hydrological stability and change in equatorial East Africa over the last 140 kyr. *Earth Planet. Sci. Lett.* **290**, 214–223 (2010).

61. L. K. Buckles, D. Verschuren, J. W. Weijers, C. Cocquyt, M. Blaauw, J. S. Sinninghe Damsté, Interannual and (multi-) decadal variability in the sedimentary BIT index of Lake Challa, East Africa, over the past 2200 years: assessment of the precipitation proxy. *Clim. Past* **12**, 1243–1262 (2016).

62. E. Gatti, A. Durant, P. Gibbard, C. Oppenheimer, Youngest Toba Tuff in the Son Valley, India: a weak and discontinuous stratigraphic marker. *Quat. Sci. Rev.* **30**, 3925–3934 (2011).

63. S. C. Jones, Palaeoenvironmental response to the∼ 74 ka Toba ash-fall in the Jurreru and Middle Son valleys in southern and north-central India. *Quatern. Res.* **73**, 336–350 (2010).

64. S. Bodé, L. De Wispelaere, A. Hemp, D. Verschuren, P. Boeckx, Water-isotope ecohydrology of Mount Kilimanjaro. *Ecohydrology* **13**, e2171 (2020).

65. S. F. Lamoureux, Embedding unfrozen lake sediments for thin section preparation. *J. Paleolimnol.* **10**, 141–146 (1994).

66. B. Masschaele, M. Dierick, D. Van Loo, M. N. Boone, L. Brabant, E. Pauwels, V. Cnudde, L. Van Hoorebeke, HECTOR: A 240kV micro-CT setup optimized for research. *J. Phys. Conf. Ser.* **463**, 012012 (2013).

67. J. Vlassenbroeck, M. Dierick, B. Masschaele, V. Cnudde, L. Van Hoorebeke, P. Jacobs, Software tools for quantification of X-ray microtomography at the UGCT. *Nucl. Instrum. Methods Phys. Res. A.* **580**, 442–445 (2007).

68. K. P. Jochum, U. Weis, B. Stoll, D. Kuzmin, Q. Yang, I. Raczek, D. E. Jacob, A. Stracke, K. Birbaum, D. A. Frick, D. Günther, J. Enzweiler, Determination of reference values for NIST SRM 610–617 glasses following ISO guidelines. *Geostand. Geoanal. Res.* **35**, 397–429 (2011).

69. K. P. Jochum, D. Scholz, B. Stoll, U. Weis, S. A. Wilson, Q. Yang, A. Schwalb, N. Börner, D. E. Jacob, M. O. Andreae, Accurate trace element analysis of speleothems and biogenic calcium carbonates by LA-ICP-MS. *Chem. Geol.* **318**, 31–44 (2012).

70. K. P. Jochum, U. Nohl, K. Herwig, E. Lammel, B. Stoll, A. W. Hofmann, GeoReM: a new geochemical database for reference materials and isotopic standards. *Geostand. Geoanal. Res.* **29**, 333–338 (2005).

71. A. Schimmelmann, H. Qi, T. B. Coplen, W. A. Brand, J. Fong, W. Meier-Augenstein, H. F. Kemp, B. Toman, A. Ackermann, S. Assonov, A. T. Aerts-Bijma, R. Brejcha, Y. Chikaraishi, T. Darwish, M. Elsner, M. Gehre, H. Geilmann, M. Gröning, J.-F. Hélie, S. Herrero-Martin, H. A. Meijer, P. E. Sauer, A. L. Sessions, R. A. Werner, Organic reference materials for hydrogen, carbon, and nitrogen stable isotope-ratio measurements: caffeines, n-alkanes, fatty acid methyl esters, glycines, L-valines, polyethylenes, and oils. *Anal. Chem.* **88**, 4294–4302 (2016).

72. P. A. Barker, E. R. Hurrell, M. J. Leng, C. Wolff, C. Cocquyt, H. J. Sloane, D. Verschuren, Seasonality in equatorial climate over the past 25 ky revealed by oxygen isotope records from Mount Kilimanjaro. *Geology* **39**, 1111–1114 (2011).

73. C. Cocquyt, E. Ryken, Afrocymbella barkeri sp. nov., (Bacillariophyta), a common phytoplankton component of Lake Challa, a deep crater lake in East Africa. *Eur. J. Phycol.* **51**, 217–225 (2016).

74. C. Cocquyt, E. Ryken, Two new needle-shaped Nitzschia taxa from a deep East African crater lake. *Diatom Res.* **32**, 465–475 (2017).

75. I. K. Seierstad, P. M. Abbott, M. Bigler, T. Blunier, A. J. Bourne, E. Brook, S. L. Buchardt, C. Buizert, H. B. Clausen, E. Cook, D. Dahl-Jensen, S. M. Davies, M. Guillevic, S. J. Johnsen,

D. S. Pedersen, T. J. Popp, S. O. Rasmussen, J. P. Severinghaus, A. Svensson, B. M. Vinther, Consistently dated records from the Greenland GRIP, GISP2 and NGRIP ice cores for the past 104 ka reveal regional millennial-scale δ18O gradients with possible Heinrich event imprint. *Quat. Sci. Rev.* **106**, 29–46 (2014).

76. S. O. Rasmussen, M. Bigler, S. P. Blockley, T. Blunier, S. L. Buchardt, H. B. Clausen, I. Cvijanovic, D. Dahl-Jensen, S. J. Johnsen, H. Fischer, V. Gkinis, M. Guillevic, W. Z. Hoek, J. J. Lowe, J. B. Pedro, T. J. Popp, I. K. Seierstad, J. P. Steffensen, A. Svensson, P. Vallelonga, B. Vinther, M. J. Walker, J. J. Wheatley, M. Winstrup, A stratigraphic framework for abrupt climatic changes during the Last Glacial period based on three synchronized Greenland ice-core records: refining and extending the INTIMATE event stratigraphy. *Quat. Sci. Rev.* **106**, 14–28 (2014).

77. L. E. Lisiecki, M. E. Raymo, A Pliocene-Pleistocene stack of 57 globally distributed benthic $\delta^{18}O$ records. *Paleoceanography* **20**, (2005).

78. D. Veres, L. Bazin, A. Landais, H. Toyé Mahamadou Kele, B. Lemieux-Dudon, F. Parrenin, P. Martinerie, E. Blayo, T. Blunier, E. Capron, J. Chappellaz, S. O. Rasmussen, M. Severi, A. Svensson, B. Vinther, E. W. Wolff, The Antarctic ice core chronology (AICC2012): an optimized multi-parameter and multi-site dating approach for the last 120 thousand years. *Clim. Past* **9**, 1733–1748 (2013).

79. M. Blaauw, B. van Geel, I. Kristen, B. Plessen, A. Lyaruu, D. R. Engstrom, J. van der Plicht, D. Verschuren, High-resolution 14C dating of a 25,000-year lake-sediment record from equatorial East Africa. *Quat. Sci. Rev.* **30**, 3043–3059 (2011).

80. P. Barker, D. Williamson, F. Gasse, E. Gibert, Climatic and volcanic forcing revealed in a 50,000-year diatom record from Lake Massoko, Tanzania. *Quatern. Res.* **60**, 368–376 (2003).

81. P. Barker, R. Telford, O. Merdaci, D. Williamson, M. Taieb, A. Vincens, E. Gibert, The sensitivity of a Tanzanian crater lake to catastrophic tephra input and four millennia of climate change. *Holocene* **10**, 303–310 (2000).

82. Y. N. Deng, P. Rioual, V. J. Jones, C. Sun, J. Mingram, A palaeoecological study investigating the impacts of multiple tephra depositions on a lacustrine ecosystem in Northeast China, using diatoms as environmental indicators. *J. Paleolimnol.* **70**, 1–22 (2023).

83. P. Roeser, N. Dräger, D. Brykała, F. Ott, S. Pinkerneil, P. Gierszewski, C. Lindemann, B. Plessen, B. Brademann, M. Kaszubski, M. Fojutowski, M. J. Schwab, M. Słowiński, M. Błaszkiewicz, A. Brauer, Advances in understanding calcite varve formation: new insights from a dual lake monitoring approach in the southern Baltic lowlands. *Boreas* **50**, 419–440 (2021).

84. J. Kalff, *Limnology: inland water ecosystems*. (Prentice Hall, New Jersey, 2002).

85. I. Meyer, M. Van Daele, N. Tanghe, M. De Batist, D. Verschuren, Reconstructing East African monsoon variability from grain-size distributions: End-member modeling and source attribution of diatom-rich sediments from Lake Chala. *Quat. Sci. Rev.* **247**, 106574 (2020).

86. M. L. Bas, R. L. Maitre, A. Streckeisen, B. Zanettin, IUGS Subcommission on the Systematics of Igneous Rocks, A chemical classification of volcanic rocks based on the total alkali-silica diagram. *J. Petrol.* **27**, 745–750 (1986).

87. N. Blegen, F. H. Brown, B. R. Jicha, K. M. Binetti, J. T. Faith, J. V. Ferraro, P. N. Gathogo, J. L. Richardson, C. A. Tryon, The Menengai Tuff: A 36 ka widespread tephra and its chronological relevance to Late Pleistocene human evolution in East Africa. *Quat. Sci. Rev.* **152**, 152–168 (2016).

88. N. Blegen, J. T. Faith, D. J. Peppe, Tephrostratigraphy of the eastern Lake Victoria Basin including the Nyanza Rift, Kenya: building a stratigraphic and chronological framework for modern human evolution. *Quat. Sci. Rev.* **256**, 106823 (2021).

89. F. Brown, B. Nash, D. Fernandez, H. Merrick, R. Thomas, Geochemical composition of source obsidians from Kenya. *J. Archaeol. Sci.* **40**, 3233–3251 (2013).

90. C. S. Lane, C. M. Martin-Jones, T. C. Johnson, A cryptotephra record from the Lake Victoria sediment core record of Holocene palaeoenvironmental change. *Holocene* **28**, 1909–1917 (2018).

91. A. Marshall, R. Macdonald, N. Rogers, J. Fitton, A. Tindle, K. Nejbert, R. Hinton, Fractionation of peralkaline silicic magmas: The greater Olkaria volcanic complex, Kenya Rift Valley. *J. Petrol.* **50**, 323–359 (2009).

92. S. C. Scott, I. P. Skilling, The role of tephrachronology in recognizing synchronous caldera-forming events at the Quaternary volcanoes Longonot and Suswa, south Kenya Rift. *Geol. Soc. Spec. Publ.* **161**, 47–67 (1999).

93. N. W. Rogers, P. J. Evans, S. Blake, S. C. Scott, C. J. Hawkesworth, Rates and timescales of fractional crystallization from 238U–230Th–226Ra disequilibria in trachyte lavas from Longonot volcano, Kenya. *J. Petrol.* **45**, 1747–1776 (2004).

94. C. A. Tryon, N. T. Roach, M. A. V. Logan, The Middle Stone Age of the northern Kenyan Rift: age and context of new archaeological sites from the Kapedo Tuffs. *J. Hum. Evol.* **55**, 652–664 (2008).

95. J. C. White, V. V. Espejel-García, E. Y. Anthony, P. Omenda, Open system evolution of peralkaline trachyte and phonolite from the Suswa volcano. *Kenya rift. Lithos* **152**, 84–104 (2012).

96. C. Martin-Jones, C. Lane, M. Van Daele, T. V. D. Meeren, C. Wolff, H. Moorhouse, E. Tomlinson, D. Verschuren, History of scoria-cone eruptions on the eastern shoulder of the Kenya–Tanzania Rift revealed in the 250-ka sediment record of Lake Chala near Mount Kilimanjaro. *J. Quat. Sci.* **35**, 245–255 (2020).

97. P. Nonnotte, M. Benoit, B. Le Gall, C. Hémond, J. Rolet, J. Cotten, P. Brunet, E. Makoba, in *Volcanism and Evolution of the African Lithosphere*. (Geological Society of America, 2011).

98. K. Fontijn, K. McNamara, A. Z. Tadesse, D. M. Pyle, F. Dessalegn, W. Hutchison, T. A. Mather, G. Yirgu, Contrasting styles of post-caldera volcanism along the Main Ethiopian

Rift: Implications for contemporary volcanic hazards. *J. Volcanol. Geotherm. Res.* **356**, 90–113 (2018).

99. V. C. Smith, N. J. Pearce, N. E. Matthews, J. A. Westgate, M. D. Petraglia, M. Haslam, C. S. Lane, R. Korisettar, J. Pal, Geochemical fingerprinting of the widespread Toba tephra using biotite compositions. *Quat. Int.* **246**, 97–104 (2011).

100. N. J. Pearce, J. A. Westgate, G. A. Gualda, E. Gatti, R. F. Muhammad, Tephra glass chemistry provides storage and discharge details of five magma reservoirs which fed the 75 ka Youngest Toba Tuff eruption, northern Sumatra. *J. Quat. Sci.* **35**, 256–271 (2020).
